# Hemocyte response to treatment of susceptible and resistant Asian corn borer (*Ostrinia furnacalis*) larvae with Cry1F toxin from *Bacillus thuringiensis*

**DOI:** 10.3389/fimmu.2022.1022445

**Published:** 2022-11-17

**Authors:** Sivaprasath Prabu, Dapeng Jing, Juan Luis Jurat-Fuentes, Zhenying Wang, Kanglai He

**Affiliations:** ^1^ State Key Laboratory for Biology of Plant Diseases and Insect Pests, Institute of Plant Protection, Chinese Academy of Agricultural Sciences, Beijing, China; ^2^ Department of Entomology and Plant Pathology, University of Tennessee, Knoxville, TN, United States

**Keywords:** *Ostrinia funacalis*, hemocyte immune function, Cry1F, C-type lecin, CD63

## Abstract

Midgut receptors have been recognized as the major mechanism of resistance to Cry proteins in lepidopteran larvae, while there is a dearth of data on the role of hemocyte’s response to Cry intoxication and resistance development. We aimed at investigating the role of circulating hemocytes in the intoxication of Cry1F toxin in larvae from susceptible (ACB-BtS) and resistant (ACB-FR) strains of the Asian corn borer (ACB), *Ostrinia furnacalis*. Transcriptome and proteome profiling identified genes and proteins involved in immune-related (tetraspanin and C-type lectins) and detoxification pathways as significantly up-regulated in the hemocytes of Cry1F treated ACB-FR. High-throughput *in vitro* assays revealed the binding affinity of Cry1F with the tetraspanin and C-type lectin family proteins. We found significant activation of MAPKinase (ERK 1/2, p38α, and JNK 1/2) in the hemocytes of Cry1F treated ACB-FR. In testing plausible crosstalk between a tetraspanin (CD63) and downstream MAPK signaling, we knocked down CD63 expression by RNAi and detected an alteration in JNK 1/2 level but a significant increase in susceptibility of ACB-FR larvae to Cry1F toxin. Information from this study advances a change in knowledge on the cellular immune response to Cry intoxication and its potential role in resistance in a lepidopteran pest.

## Introduction

The bacterium *Bacillus thuringiensis* (Bt-) produces insecticidal Cry proteins, some of them presenting specificity against lepidopteran larvae (1). Upon ingestion, Cry proteins bind to receptors and form pores on the brush border membrane of midgut cells, resulting in epithelial disruption and passage of gut bacteria into the main body cavity to cause lethal septicemia (2). Selected *cry* genes were transformed into plants to develop and commercialize transgenic crops producing Cry proteins to control economically relevant lepidopteran pests, especially stem borers that are difficult to control with sprayed pesticides (3). The efficacy and benefits of this technology are clearly exemplified by adoption of corn-producing the Cry1Ab protein against the European corn borer, *Ostrinia nubilalis*, which resulted in areawide population suppression (4) and extended pest control benefits to vegetable growers (5). Based on this success, it is expected that transgenic corn expressing Cry1 genes could control larvae of the Asian corn borer (ACB), *Ostrinia furnacalis*, one of the most relevant pests of corn in Asia (6). However, resistance to various Bt proteins, including Cry1Ab, Cry1Ac, Cry1Ah and Cry1F, has been observed after laboratory selection of ACB strains (7–9).

Given that Cry proteins target midgut cells in the host insect, most research efforts have focused on midgut alterations for mechanistic characterization of resistance to Cry proteins (10). In contrast, the role of cellular immunity in response to Cry intoxication and resistance has been rarely explored. Circulating hemocytes are a vital constituent of hemolymph in lepidopteran larvae and have fundamental roles in detoxifying toxic complexes and cellular defense against pathogenic microbes (11, 12). Hemocytes express pattern recognition receptors (PRRs) on their surface that recognize foreign bodies and humoral macromolecules to activate intracellular effector molecules triggering appropriate defensive responses, including phagocytosis, nodulation, and encapsulation (12, 13). Among the relevant intracellular factors modulating hemocyte activity are members of the mitogen-activated protein kinase (MAPK) family, including extracellular signal-regulated kinases (ERKs), c-Jun N-terminal kinase (JNK) and p38 (14). There is emerging evidence in Lepidoptera of a relevant role for MAPKs in modulating the response to Cry intoxication. For instance, knockdown of the *p*38 gene significantly increased susceptibility to Cry1Ca in *Chilo suppressalis* (15) and Cry1Ab in *Manduca sexta* (16). In *Plutella xylostella*, a hormone-activated MAPK signaling pathway reduced the expression of midgut genes encoding receptors for the Cry1Ac protein, resulting in resistance (17). Further work determined that this MAPK signaling cascade is modulated by 20-hydroxyecdysone and juvenile insect hormones, probably allowing for lack of fitness costs that are often associated with other resistance mechanisms (18). Recently, the genes involved in this Cry1Ac-induced MAPK response have been identified (19). Another relevant protein for hemocyte defensive function that may participate in response to Cry intoxication is tetraspanin (20). Specifically, a field-derived dominant mutation in a tetraspanin gene was linked with resistance to Cry1Ac in the cotton bollworm, *Helicoverpa armigera* and may explain the faster evolution of field-evolved resistance in this insect (21).

In ACB, alterations affecting midgut receptors were reported in ACB resistant to Cry1Ab and Cry1Ac (22, 23), while resistance mechanisms to Cry1F and Cry1Ah are less clear. Transcriptomic and proteomic analyses in ACB resistant to Cry1Ah detected down-regulation of putative receptor and detoxification genes, while anti-stress genes were up-regulated (24). Similar studies in ACB resistant to Cry1Ie or Cry1F detected down-regulation of multiple genes when compared to susceptible insects (25). Notably, the Cry-1F resistant ACB-FR strain presented up-regulation of glutathione-S-transferase, which was not detected in Cry1Ie-resistant ACB.

In this study, we aimed to test the potential participation of cellular immunity in resistance to Cry1F by comparing hemocyte gene expression after treatment with Cry1F in susceptible and Cry1F-resistant ACB larvae. Several differentially expressed defense-related genes were identified through a combined next-generation sequencing and proteomics approach. The interactions between Cry1F and proteins encoded by these differentially expressed genes and their functional role were tested by surface plasmon resonance and RNA interference assays. Results provide evidence for interactions between Cry1F and hemocyte proteins that may be relevant in determining susceptibility to the toxin.

## Materials and method

### Insecticidal protein

Trypsin-activated Cry1F toxin was purchased from Envirologix (Portland, OR, USA).

### Insects

Susceptible (ACB-BtS) and Cry1F-resistant (ACB-FR) strains of ACB were described previously (8) and were reared on an artificial diet as reported elsewhere (26). Susceptibility estimates for Cry1F toxin (LC_50_ value, Cry1F concentration killing 50% of ACB larvae) were determined from 7-day diet incorporated bioassays with ACB neonates as 1.2 μg/g and 1,075 μg/g (toxin/diet) for the ACB-BtS and ACB-FR strains, respectively. The results indicated more than 903-fold resistance in ACB-FR.

### Hemocyte and hemolymph samples

Hemolymph was collected from 4^th^ instar ACB-BtS and ACB-FR larvae after exposure to their respective Cry1F LC_50_ values (1.2 μg/g for ACB-BtS and 1,075 μg/g for ACB-FR) for 24 and 48 hours. Larvae exposed to an untreated diet were used as controls. Hemolymph was collected after surface sterilizing the ventral surface of the abdomen using 75% ethanol and cutting a proleg with fine scissors. About 10 μL of hemolymph, containing around 1.9 × 10^6^ hemocytes/mL as determined by counting in the hemocytometer chamber, were collected per larva. Hemolymph collected from 50 larvae was pooled as a biological replicate in 200 μL of ice-cold iso-osmotic Tris-buffered saline (ITBS, 50 mM Tris-HCl, 10 mM CaCl_2_, 100 mM NaCl, 100 mM dextrose, 6 mM KCl, 5 mM MgCl_2_, pH 7.2, 300 mOsm) to prevent hemocyte aggregation [27]. Three biological replicates were prepared and processed per ACB strain. The hemocyte suspensions were centrifuged (400 × *g*, 15 min, 4°C), and the resultant hemocyte pellets were used for RNA extraction and RNA-Seq analysis. The cell-free hemolymph in the supernatants was used for iTRAQ analysis.

### RNA extraction and cDNA synthesis

Total RNA was extracted from hemocytes using TRIzol^®^ (Ambion, Life Technologies, CA, USA), following the manufacturer’s instructions. The RNA concentration and quality were evaluated using a NanoDrop 2000 spectrophotometer (Thermoscientific, MA, USA). Complementary DNA (cDNA) was synthesized from 700 ng of total RNA using the One-Step gDNA Removal and cDNA Synthesis SuperMix kit (TransGen Biotech Co., Ltd., Beijing, China), following the manufacturer’s protocol.

### RNA-Seq library preparation, sequencing and analysis

Libraries were prepared from 1 μg of total RNA per sample using the NEBNext^®^ Ultra™ RNA Library Prep Kit for Illumina (New England Biolabs, MA, USA), according to the manufacturer’s instructions. Briefly, mRNA was purified from total RNA using magnetic beads coated with poly-T oligo, and after fragmentation was used for cDNA synthesis and ligation of adaptors. cDNA library fragments of 250-300 bp in length were purified with the AMPure XP system (Beckman Coulter, MA, United States) and then used for PCR with Universal and Index (X) PCR primers. Finally, PCR products were purified (AMPure XP system), and library quality was assessed on the 2100 Bioanalyzer (Agilent, CA, USA). The clustering of the index-coded samples was performed on a cBot Cluster Generation System using TruSeq PE Cluster Kit v3-cBot-HS (Illumina, CA, USA), according to the manufacturer’s instructions. After cluster generation, the libraries were sequenced on a Novaseq 6000 platform at Novogene Co. Ltd. (Beijing, China) with a 150 bp paired-end sequencing configuration.

Raw reads were processed to clean reads through custom Perl scripts by removing adapter-related sequences (796,654, 3.45%), ploy-N (23,878, 0.10%) and low-quality reads (42,988, 0.19%). A publicly available ACB genome assembly (Version: ASM419383v1, GenBank accession: GCA_004193835.1) was downloaded and used as the reference genome for assembly reads. Paired-end clean reads were aligned to the reference genome using Hisat2 v2.0.5 (27). Differential expression analysis was performed using the DESeq2 R package v1.16.1 (28). The resulting *P*-values (*P*<0.05) were adjusted using Benjamini and Hochberg’s approach for controlling the false discovery rate. KEGG statistical (*P*<0.05) enrichment of differentially expressed genes was performed using the cluster Profiler v3.12 R package (29). The raw data from the three biological replicates is publicly available in the Sequence Read Archive (SRA) database as BioProject ID PRJNA723187.

### Quantification of gene expression (qRT-PCR)

Primers were designed to amplify selected target genes (**Table S1**, Supporting Information), and specificity was confirmed by amplification and sequencing of amplicons (**Figure S1**; **Supplementary file**). Original cDNA samples were diluted 10-fold, five serially diluted concentrations were subjected to qRT-PCR, and mean CT values from three replicates were used to construct the standard curve. The amplification efficiency (E value) of all primers was calculated using the formula: E = 10^(−1/slope)^ -1 (30). A two-step PCR approach was implemented using 20 μL final reaction volumes in the TB Green™ mix. Amplification by qRT-PCR was performed on an ABI 7500 Fast (Applied Biosystems, USA) thermocycler with TB Green™ Premix Ex Taq™ (TaKaRa, Japan). The amplification program was set as follows: 1) initial denaturation 30 s at 95°C, 2) 95°C for 5 s, and 3) 60°C for 30 s; steps 2 and 3 were repeated for 40 cycles. Ribosomal protein L8 was used as a reference gene (31), and the 2^-ΔΔCT^ method was used to calculate the difference in expression of selected genes (32). Three independent biological replicates were performed per treatment, and three technical replicates were performed per biological sample.

### Glutathione S-transferase activity

The GST activity was estimated in hemolymph from larvae of the ACB-BtS and ACB-FR strains after 24 and 48 hours of treatment with Cry1F (LC_50_ dose) or a control diet. The protein concentrations in the samples were estimated using the Easy Protein quantitative kit (TransGen Biotech Co., Ltd., Beijing, China) and diluted to 1 mg/mL using the 1 × GST sample buffer provided in the kit. The activity of GST was measured using the Glutathione S-Transferase Assay Kit (Cayman Chemical, MI, USA), according to the manufacturer’s instructions. Briefly, 20 µL of hemocyte-free hemolymph was added to individual wells in a 96-well microplate, and then 150 µL of assay buffer and 20 µL of glutathione provided with the kit were added per well. Finally, 1-chloro-2,4-dinitrobenzene (CDNB) was added (10 µL), and the plate was incubated for 10 min at RT and then read at 340 nm to measure the conjugation of CDNB with reduced glutathione.

### Hemolymph proteomic analysis

Isobaric tag for relative and absolute quantitation (iTRAQ) proteomic analysis was conducted as described elsewhere (24) on hemolymph proteins from ACB-BtS and ACB-FR larvae treated with Cry1F (LC_50_ dosage). Briefly, the hemolymph protein samples were precipitated with acetone and digested using sequencing-grade modified trypsin. The digested peptides were labeled with iTRAQ reagents and analyzed by LC-MS/MS for identification and quantification. The hypergeometric distribution equation analyzed the significantly enriched proteins in the KEGG pathway (*P*< 0.05). Gene Ontology classification analysis was performed using OmicsBox v1.2.4 integrated with the Blast2GO function annotation module (33). The enrichment of the differentially expressed proteins was represented in the -LOG_10_ *P*-value.

### Immune-related protein selection and in silico protein-protein interaction analysis

Proteins selected for PPI were macrophage mannose receptor 1-like (MMR1), C-type mannose receptor 2-like (C-MR2), CD209 antigen-like protein E (CD209), C-type lectin lectoxin-Phi1-like (C-lectoxin), hemolymph lipopolysaccharide-binding protein (LBP), and CD63 antigen-like (CD63). The predicted full-length amino acid sequences of activated Cry1F and the hemocyte proteins selected for further studies were subjected to structure homology modeling (**Supplementary file**) (Swiss-Model server, https://swissmodel.expasy.org/) (34). The TMHMM Server v2.0 (http://www.cbs.dtu.dk/services/TMHMM/) (35) was employed for transmembrane prediction analysis. The protein domain prediction was achieved by analyzing all the sequences using the Pfam or InterProScan database (http://pfam.xfam.org/) (36) (https://www.ebi.ac.uk/interpro/) (37). The DOG 2.0 software was used to illustrate the domains of selected hemocyte proteins and Cry1F (38). The *in silico* protein-protein interaction (PPI) was analyzed using carbohydrate-binding domains of C-type lectins and the extracellular domain (EC 2) of tetraspanin (CD63) with the central β-sheet domain of Cry1F. The solvent-accessible surface-exposed residues in the interacting domains were predicted by RaptorX-Property Prediction tool (http://raptorx.uchicago.edu/) (39). The domain-based PPIs were carried out using the High Ambiguity Driven protein-protein DOCKing approach (HADDOCK 2.4 server) (40). The HADDOCK 2.4 default settings were used, and no special constraints were input during the PPI studies. The UCSF ChimeraX v1.1 was used to visualize the PPI complex after the *in silico* analysis (41).

### ACB hemocyte protein expression and purification

The full length cDNAs encoding selected C-type lectins and tetraspanin and including a N-6 His-Tag were synthesized using a PCR-based Accurate Synthesis (PAS) system. These synthesized protein-coding ORFs were constructed into the pCZN1 vector at EcoRv-XbaI sites and expressed in the ArcticExpress Competent Cells *E. coli* expression system (Agilent Technologies, CA, USA). Transformants were grown in liquid LB medium to OD 0.6-0.8 and then induced by the addition of 1 mM isopropyl-b-d-thiogalactoside (IPTG, Coollaber Science and Technology, Beijing, China) and placed in a shaking incubator at 37°C for 6 h. The induced competent cells were harvested by centrifugation (8,000 × *g*, 4°C, 15 min) and the pellets were resuspended in 20 mL of lysis solution (20 mM Tris-HCL, 1 mM phenylmethylsulfonyl fluoride, bacterial protease inhibitor cocktail, pH 8.0). The induced competent cells resuspension was subjected to ultra-sonication (400 W, operating 4 sec, paused 8 sec, total duration 20 min). The ultra-sonicated cell lysate solution was centrifuged (10,000 × *g*, 4°C, 15 min) and the pelleted inclusion bodies washed in washing solution (20 mM Tris-HCL, 1 mM EDTA, 2 M carbamide, 1 M NaCl, 1% Triton X-100, pH 8.0). These centrifugation and washing steps were performed a total of three times and then inclusion bodies in washing solution were solubilized in a 1:2 (v/v) ratio of lysis buffer (20 mM Tris-HCL, 5 mM DTT, 8 mM carbamide, pH 8.0) overnight at 4°C. After the overnight incubation, the His-tag protein precipitate was recovered by centrifugation (10,000 × *g*, 4°C, 15 min), resuspended and dialyzed overnight in Tris buffer (20 mM Tris-HCL, 0. 15 mM NaCl, pH 8.0).

The recombinant proteins were purified in a Ni-IDA-Sepharose Cl-6B affinity column coupled to a low-pressure chromatography system (BioLogic LP System with BioFrac Fraction Collector, Bio-Rad, CA, USA) at a flow rate of 1 mL/min, using 20 mM Tris-HCL with 250 mM imidazole (pH 7.4) for elution. Proteins in eluted fractions were resolved by 12% SDS-PAGE and transferred (100 V, 200 mA, 1 h) to a polyvinylidene fluoride (PVDF) filter. After transfer, the filter was washed four times in phosphate-buffered saline with Tween-20 (PBST) and then blocked for 1 hour in PBST plus 5% skim milk. The PVDF filter was then incubated in primary rabbit antisera against the His-tag (1:1,000 dilution) on a shaker overnight at 4°C. After washing in PBST, the filter was probed with sheep anti-rabbit secondary antibody conjugated to horseradish peroxidase (HRP, 1:10,000 dilution) at 37°C for 1 h. The filter was then washed once before developing using enhanced chemiluminescence (ECL Western Blotting Substrate, Pierce, USA) in an ImageQuant LAS 4000 (GE Healthcare, PA, USA) imager.

Purified MMR1, C-MR2, C-lectoxin, LBP and CD63 proteins (1 mg) to be used for SPR analysis were digested using 5U of SUMO Protease (Beijing Solarbio Science & Technology Co., Ltd, China) in PBS (pH 7.2) at 4°C overnight to remove the N-6xHis tag, as confirmed by Western blotting. Samples of purified MMR1, C-MR2, C-lectoxin, LBP and CD63 proteins non-digested with SUMO Protease were subjected to immobilized-metal affinity chromatography (IMAC) using a nickel Ni-NTA matrix (Beijing Solarbio Science & Technology Co., Ltd, China). The purified proteins containing the N-6xHis tag were used as bait proteins in the pull-down assays, while proteins purified without the His-tag were used in surface plasmon resonance analysis to avoid interference with PPI analyses.

### Surface plasmon resonance analysis

Macromolecular interactions between Cry1F protein and C-type lectins or tetraspanin were analyzed on a Biacore™ 8K surface plasmon resonance system (GE Healthcare, IL, USA). The activated Cry1F activated toxin (0.5 mg/mL) was immobilized on a CM5 (GE Healthcare, IL, USA) sensor chip using sodium acetate (10 mM, pH 4.0) as immobilization buffer with a flow rate of 10 μL/min. Running and regeneration buffers were HEPES (10 mM HEPES, 150 mM NaCl, 3 mM EDTA, 0.005% Tween-20, pH 7.4) and 3M magnesium chloride (pH 7.4), respectively. The association (120 sec) and disassociation (280 sec) steps were maintained at the flow rate of 30 μL/min. The analytes (MMR1, C-MR2, C-lectoxin, LBP, CD209 and CD63 without His-Tag) were desalted using the running buffer before SPR analysis. To calculate the equilibrium dissociation constant, the analytes that could interact with Cry1F were used to perform multiple analyte concentration injections. The sensogram data sets were fit to 1:1 binding model to determine binding parameters.

### Pull-down assays

Pull-down assays were performed using the Pierce™ Pull-Down PolyHis Protein : Protein Interaction Kit (Thermo Scientific, USA), according to manufacturer’s instructions. The MMR1, C-MR2, C-lectoxin, LBP, and CD63 proteins containing a N-6 His-Tag were immobilized on HisPur™ Cobalt Resin as bait proteins. Initially, 50 μL of HisPur™ Cobalt Resin was used to separately immobilize the MMR1, C-MR2, C-lectoxin, LBP, and CD63 proteins. About 300 μL of protein sample (0.4 mg/mL) were added to each of the columns containing cobalt resin, and the mixtures were incubated for 1 hour at 4°C on a rocking platform. After incubation, the columns were placed on a new collection tube and spun down (1,200 × *g* for 1 min at RT) to eliminate the flowthrough. The columns were subsequently washed with buffer and centrifuged five times to eliminate unbound bait proteins. After washing, 600 μL of activated Cry1F toxin (prey protein, 0.3 mg/mL) were added to each column, and after sealing the columns were incubated for 2 h at 4°C on a rocking platform. After incubation, the columns were placed on a new collection tube and spun down (1,200 × g for 1 min at RT) to collect and discard the flowthrough, The columns were washed five times to eliminate the residue of prey protein that did not interact with bait protein. The bait and prey column flowthroughs were later used for comparison by SDS-PAGE analysis. For elution of interacting prey and bait proteins, 200 μL of elution solution (290 mM imidazole) was added to the columns, which were then sealed and incubated for 20 min at RT on a rocking platform. The final eluate from each column was collected by centrifugation (1,200 × g for 1 min at RT) in a new collection tube. A non-treated control with cobalt resin without immobilized bait proteins was also run to identify and eliminate false positives caused by nonspecific binding of proteins to the HisPur™ cobalt resin. The final elution was analyzed in a 4-12% ExpressPlus™ PAGE gel (GenScript, USA) using 15 μL of eluate. Proteins were detected by staining with the FastBlue Protein stain (Coolaber, Beijing, China) and ProteoSilver silver staining kit (Sigma-Aldrich, MA, USA), according to the manufacturer’s instructions.

### MAPK activity

Activated Cry1F toxin at the LC_50_ concentration (1.2 μg/g for ACB-BtS and 1,075 μg/g for ACB-FR) was incorporated in an artificial diet and used to feed 4^th^ instar susceptible and Cry1F-resistant ACB larvae. After 48 hours, the hemolymph was collected, and hemocytes isolated as described above. The pelleted hemocytes were washed three times with iso-osmotic tris buffered saline (ITBS, pH 7.2; 300 mOsm). The hemocyte lysate sample and MAPK family assays were prepared using the InstantOne™ MAPK Family Activation Multispecies ELISA Kit (Thermofisher Scientific, MA, USA), according to the manufacturer’s instructions. The kit consists of antibody cocktails for detecting three important types of protein kinase; extracellular signal-regulated kinases 1/2 (ERK 1/2), MAPK p38α and c-Jun N-terminal kinase 1/2 (JNK 1/2). The protein concentrations in the lysate samples were estimated using the Easy Protein quantitation kit (TransGen Biotech Co., Ltd., Beijing, China). Before performing the assays, the final concentration of cell lysate samples was set to 0.5 mg/mL. Three biological replications (hemolymph from 30 pooled larvae/replicate) were performed per sample.

### Functional testing CD63 for participation in resistance to Cry1F

We prepared double stranded RNA (dsRNA) targeting the CD63 gene (dsCD63) and orally delivered it to ACB-FR. Briefly, gene-specific primers (**Table S1**, Supporting Information) containing a T7 promoter on the 5’ end were designed using the Primer Premier 5.0 software (Premier Biosoft, Canada) and used to amplify CD63 from a plasmid containing the CD63 ORF (pCloneEZ) or the green fluorescent protein (GFP) ORF from vector pRTHSP70-GFP containing the GFP gene (GenBank accession number AB062168.1) Amplicons (572 bp for CD63 and 467 bp for GFP) were used as templates for *in vitro* dsRNA synthesis using the T7 RiboMAX Express RNAi System (Promega, Madison, WI, USA), following the manufacturer’s instructions.

Fourth instar ACB-FR larvae were starved for 6 hours and then fed a control diet or diet containing Cry1F at the LC_50_ concentration and dsRNA targeting the GFP or CD63 genes (1 µL containing 700 ng per mg of diet). After the larvae ate the diet pieces, fresh artificial diet incorporated with toxin (without dsRNA) was added. Three independent replicates (24 larvae pooled/replicate) were maintained. After 8 hours, the second dose of dsGFP or dsCD63 was added to the diet in order to sustain the silencing effect. The hemolymph was collected from larvae after 24, 48, 72, 96 and 120 hours, and hemocytes recovered as described above. RNA was extracted from hemocytes collected at different time points, three biological replicates were used for each time point (hemolymph from 30 larvae pooled/replicate). The level of MAPK activity was assessed in the hemocytes of dsRNA treated and control groups after 48 hours, three biological replicates were performed for each treatment (hemolymph from 30 larvae pooled/replicate). Purified RNA was used to prepare cDNA using the cDNA Synthesis SuperMix (TransGen Biotech Co., Ltd., Beijing, China). This cDNA was used as a substrate in qPCR analyses using the TB Green™ Premix Ex Taq™ (TaKaRa, Japan) to detect transcript levels of the CD63 gene. Relative expression levels were estimated with the 2^-ΔΔCT^ method [32] using transcript levels for RPL8 (GenBank Acc. No. XM_028306632.1) as an internal reference gene. Control treatment with a diet not containing dsRNA or Cry1F toxin was used for normalization of CD63 gene expression.

### Statistical analyses

Student’s *t*-test (*P* < 0.05) was used to analyze GST activity and qRT-PCR expression data. One-way ANOVA (*P* < 0.05) was used to test for significant differences among treatments for other experiments. Differences in MAPK levels among hemocytes of ACB-BtS and ACB-FR after exposure to Cry1F toxin were compared with their respective control groups. Percentage larval mortality data from the RNAi experiment was transformed using Arcsine square root and treatment means were analysed using One-way ANOVA (Tukey test LSD, *P*< 0.05). All analyses were performed using IBM SPSS v 20 (IBM Corporation, Armonk, NY).

## Results

### Differential gene expression in hemocytes after Cry1F treatment

Differentially expressed genes (DEGs) were identified in hemocytes from larvae of the ACB-BtS and ACB-FR strains after treatment for 48 h with their respective LC_50_ concentrations of Cry1F when compared to expression in hemocytes from larvae treated with control diet for each strain (**Figure S2**; **Supplementary file**). Treatment of ACB-FR larvae with Cry1F induced up-regulation of 4,289 genes, while only 890 genes were up-regulated in larvae from the ACB-BtS strain after toxin treatment (**Figure S2**; **Supplementary file**). On the other hand, Cry1F treatment induced down-regulation of 4,532 genes in ACB-FR and 975 in the ACB-BtS strains (**Figure S2**; **Supplementary file**).

**Figure 1:**
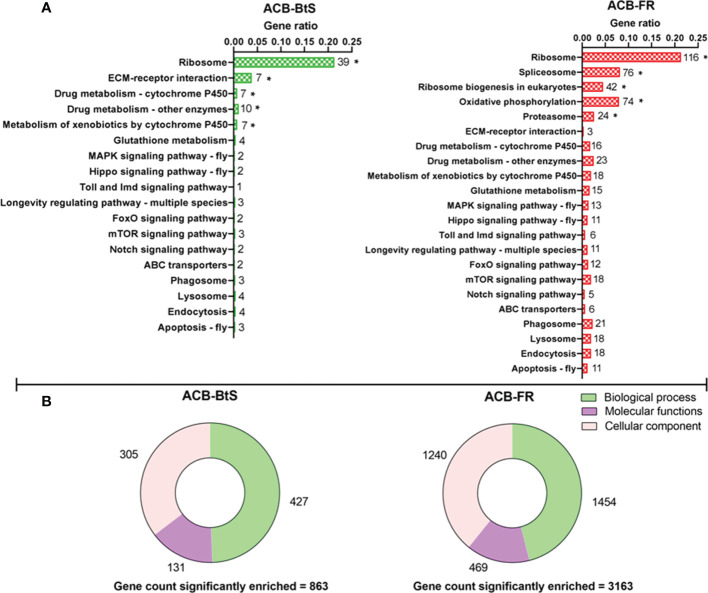
Differential gene expression after Cry1F treatment in hemocytes from susceptible (ACB-BtS) and Cry1F-resistant (ACB-FR) strains of Asian corn borer. **(A)** Hemocyte sample processing and enriched genes grouped after KEGG analysis. The numbers shown on the right side of every horizontal bar represent the number of genes involved in that specific pathway. Asterisks represent significant enrichment of genes in the hemocytes of Cry1F- treated ACB-BtS and ACB-FR larvae compared with respective non-treated controls (Student’s *t*-test, *P*<0.05). **(B)** Gene Ontology analysis shows the number of significantly enriched genes in the hemocytes of Cry1F treated ACB-BtS and ACB-FR larvae compared to respective non-treated controls (Student’s t-test, *P*<0.05).

Overall, the KEGG pathway analysis detected enrichment for both ACB-BtS and ACB-FR in genes involved in direct/indirect control of cellular activities such as adhesion, migration, differentiation, proliferation, and apoptosis. In ACB-BtS, significant enrichment was detected for 70 genes involved in 5 different pathways, including Ribosome, ECM-receptor interaction, Drug metabolism - cytochrome P450, Drug metabolism - other enzymes and Metabolism of xenobiotics by cytochrome P450 (**Figure 1A**). In contrast, the 332 genes significantly enriched in ACB-FR were assigned in Ribosome, Spliceosome, Ribosome biogenesis in eukaryotes, Oxidative phosphorylation and Proteasome pathways (**Figure 1A**). Gene Ontology (GO) analyses identified 863 genes significantly enriched in ACB-BtS hemocytes after Cry1F treatment, while in ACB-FR that number increased to 3,163 genes. Interestingly, the relative proportion of enriched genes in each category (biological process, molecular function and cellular component) was significantly higher in ACB-FR strains (**Figure 1B**).

**Figure 2:**
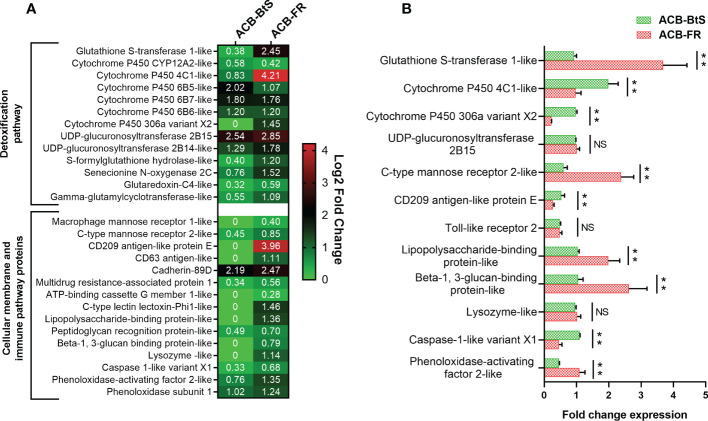
Analysis of differentially expressed genes encoding membrane and immune/detoxification proteins in hemocytes after Cry1F treatment in susceptible (ACB-BtS) and Cry1F-resistant (ACB-FR) strains of Asian corn borer. **(A)** Differentially expressed genes (DEGs) and fold change compared with respective controls (Student’s t-test, *P*<0.05). The numbers in the heat map represent the log2 fold change in gene expression, a value of 0 indicates that the gene was not detected in the DEGs or down-regulated lists. **(B)** Difference (fold change) in expression levels (mean ± standard error) of genes involved in detoxification and immune pathways of hemocytes from the ACB-BtS and ACB-FR strains after Cry1F treatment (LC50 dose). Asterisks denote that differences in gene expression were statistically significant (Student’s t-test, **P<0.01, NS, not significant).

Based on Cry1F receptors being membrane proteins and the potential participation of the immune system in response to intoxication and potentially resistance, we constructed a heat map of up-regulated genes encoding cellular membrane proteins and proteins involved in detoxification and immune-related pathways (**Figure 2A**). The biggest differences in expression detected after feeding on Cry1F compared to control diet in both ACB-BtS and ACB-FR strains were observed for UDP-glucuronosyltransferase 2B15 (2.54- and 2.85-fold in ACB-BtS and ACB-FR, respectively), and cadherin 89D (2.19- and 2.47-fold in ACB-BtS and ACB-FR, respectively). Genes that were highly up-regulated in ACB-FR but not in ACB-BtS included gluthathione S-transferase (2.45- versus 0.38- fold in ACB-BtS), cytochrome P450 4C1-like (4.2- versus 0.83-fold in ACB-BtS), and CD209 antigen-like protein E (3.96-fold in ACB-FR and not detected among DEGs in ACB-BtS). Other DEGs unique to ACB-FR were CD63 antigen-like, lysozyme-like and C-type lectin lectoxin-Phi1-like (**Figure 2A**).

Differential expression of selected genes involved in detoxification and immune pathways between ACB-BtS and ACB-FR was tested using qRT-PCR (**Figure 2B**). Compared to ACB-BtS, treatment with Cry1F induced significantly higher expression in ACB-FR hemocytes of GST (3.68-fold), C-type mannose receptor 2-like (2.38-fold), lipopolysaccharide-binding protein-like (1.98-fold), beta-1,3-glucan-binding protein-like (2.62-fold) and phenoloxidase-activating enzyme-like (1.09-fold). On the other hand, cytochrome P450 4C1-like, cytochrome P450 306a variant X2, CD209 antigen-like protein E and caspase 1-like gene had significantly higher expression in ACB-FR in RNAseq but had lower transcript levels when compared to ACB-BtS in qRT-PCR (**Figure 2B**).

**Figure 3:**
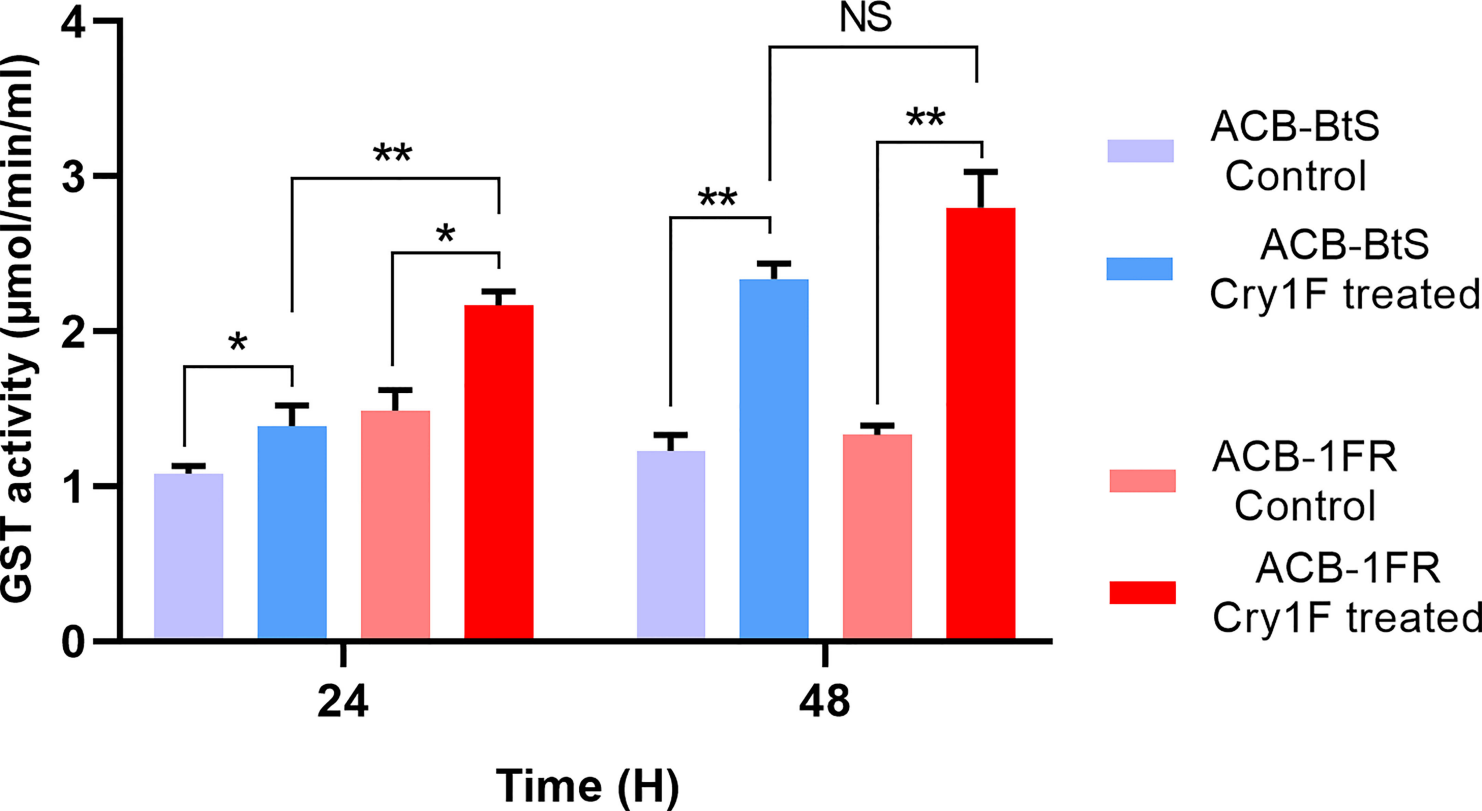
Glutathione S-transferase (GST) activity in hemolymph of susceptible and resistant Asian corn borer (ACB) larvae after exposure to Cry1F (LC_50_ dosage: ACB-BtS - 1.2 μg/g and ACB-FR 1,075 μg/g, toxin/diet). Asterisk represents statistically significant difference (Student’s t-test, *P<0.05, **P<0.01), NS, not significant.

Based on the consistent high GST expression difference detected, we performed GST enzymatic assays comparing hemolymph from ACB-BtS and ACB-FR larvae. Treatment with Cry1F for 24 and 48 hours increased GST activity in hemolymph from larvae of both strains (**Figure 3**). In agreement with RNAseq and qRT-PCR results, GST activity was clearly higher in hemolymph from ABC-FR compared to ACB-BtS. This difference in GST activity between the strains was statistically significant after 24 but not after 48 hours of treatment with Cry1F (**Figure 3**).

### Differential levels of hemolymph proteins after Cry1F treatment

**Figure 4:**
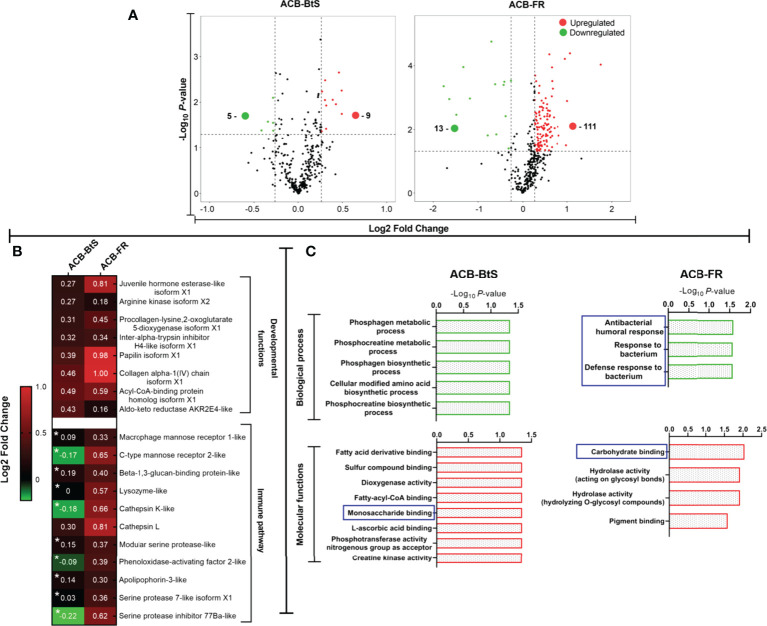
Differential protein levels detected in hemolymph from ACB-BtS and ACB-FR larvae after treatment with Cry1F. **(A)** Hemolymph sample processing and Volcano plot showing the number of proteins with significantly increased (red dots) or reduced (green dots) abundance in the hemolymph of Cry1F-treated ACB-BtS and ACB-FR larvae compared to controls (Student’s t-test, *P*<0.05). **(B)** Differentially abundant proteins (DAPs) in hemolymph from Cry1F treated ACB-BtS and ACB-FR larvae involved in developmental functions and immune pathways. Asterisks in the ACB-BtS column indicate proteins with no significant change in abundance (Student’s t-test, *P*<0.05). A value of 0 indicates the protein was not detected in the DAPs list. **(C)** Enriched (gene ontology) hemolymph proteins according to biological process and molecular functions in hemocytes from ACB-BtS and ACB-FR larvae after Cry1F treatment. Pathways and respective functions marked in a blue box indicate enrichment of proteins with immune function (-Log10, P-value).

Analysis by iTRAQ identified differentially abundant proteins (DAPs) in the hemolymph of ACB larvae after Cry1F treatment compared to controls (**Figure 4A**). Comparatively, a much higher number of DAPs with increased abundance were detected in the hemolymph of ACB-FR (111 proteins) compared to ACB-BtS (9 proteins). Among DAPs involved in immune pathways, all but one had a higher abundance in hemolymph from ACB-FR compared to ACB-BtS larvae (**Figure 4B**). Importantly, DAPs included DEGs such as macrophage mannose receptor 1-like (MMR1), C-type mannose receptor 2-like (C-MR2), beta-1 3-glucan-binding-like, lysozyme-like and phenoloxidase-activating factor 2-like (**Figure 2A**). Cathepsin L was the most significantly enriched protein in hemolymph of both strains and was identified as involved in phagosome/autophagy pathways (**Figure 4B**). Gene ontology (GO) annotation of iTRAQ data showed that ACB-BtS biological processes were related to metabolism, while ACB-FR were related to antibacterial defense mechanisms (**Figure 4C**). Carbohydrate-binding or recognition protein was a molecular function commonly enriched in both ACB-BtS and ACB-FR (**Figure 4C**).

### In silico and *in vitro* interactions between Cry1F and hemolymph proteins

Based on commonalities in results from RNA-Seq, qRT-PCR and iTRAQ analyses, DEGs and DAPs involved in defense response to bacteria, carbohydrate-binding, and a transmembrane protein involved in cellular signaling were selected for *in silico* protein-protein interaction (PPI) studies. Selected proteins included members of the tetraspanin superfamily (CD63 antigen-like), a hemolymph lipopolysaccharide-binding protein (LBP), the CD209 antigen-like protein E (CD209), and C-type lectins macrophage mannose receptor 1-like (MMR1), mannose receptor 2-like (C-MR2), and lectoxin-Phi1-like (C-lectoxin). All the proteins were annotated in UniProtKB and QuickGo databases with their putative functions (**Table 1**).

**Table 1:** Putative functions annotated for hemocyte proteins involved in immune-related functions.

Family(Pfam)	Hemocyte proteins	UniProtKB(General function & Identifier)	QuickGo Gene ontology		
			Biologicalprocess	Molecularfunction	Subcellular location
C-type lectin	MMR1	Endocytosis of glycoproteins by macrophages (P22897)	Cellular response to lipopolysaccharide (GO:0071222)	Mannose binding (GO:0005537)	Plasma membrane Endosome
(PF00059.21)	C-MR2	Internalizes glycosylated ligands (Q9UBG0)	Endocytosis (GO:0006897)	Signalling receptor activity (GO:0038023)	Plasma membrane
	CD209	Putative pathogen-recognition receptor. (Q91ZW7)	Endocytosis (GO:0006897)	Mannose binding (GO:0005537)	Plasma membrane
	C-lectoxin	Mannose-binding lectin (A7X406)	AN	Carbohydrate binding (GO:0030246)	Extracellular region or secreted
	LBP	Trapping intracellular symbionts (P26305)	AN	Carbohydrate binding (GO:0030246)	Extracellular region or secreted
Tetraspanin (PF00335.20)	CD63	Activation of integrin signalling and cascading activation of AKT, FAK/PTK2 and MAP kinases (P41731)	Positive regulation of receptor internalization (GO:0002092)	Positive regulation of endocytosis (GO:0045807)	Plasma membrane, endosome, lysosome and nucleus

AN, Annotation not available.

**Figure 5:**
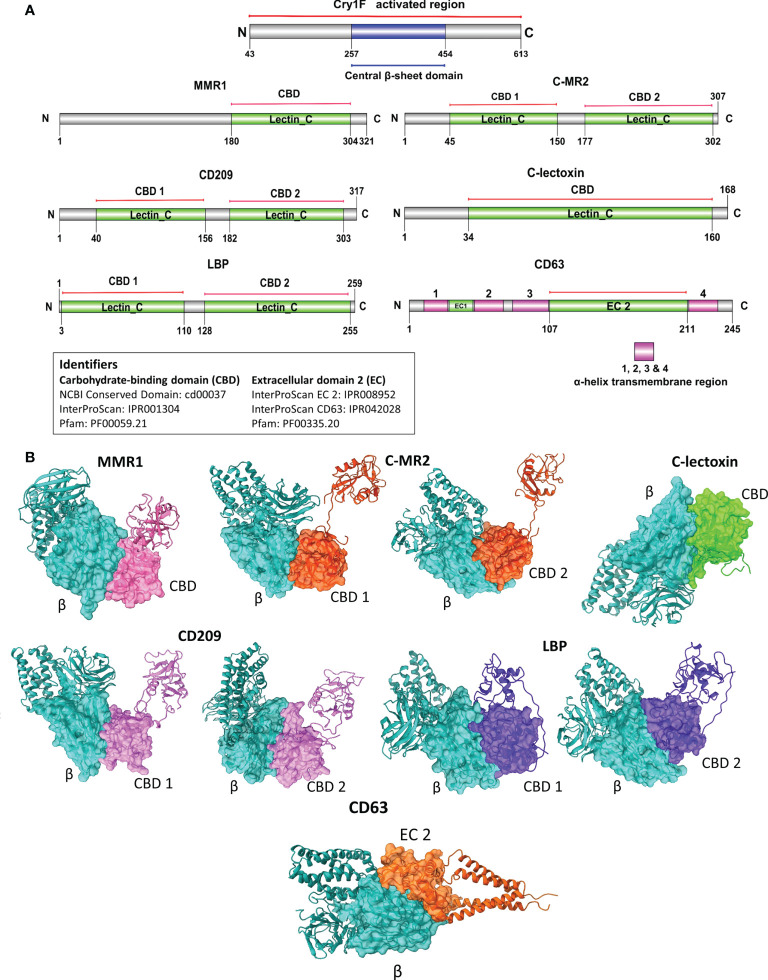
Domain-based protein-protein interaction (PPI) *in silico* analysis of C-type lectins and tetraspanin with domain II of Cry1F. **(A)** Protein domains identified in C-type lectins, tetraspanin and Cry1F by NCBI Conserved domain, Pfam or InterProScan. **(B)** The PPI complexes of Cry1F with C-type lectins and tetraspanin (β = Central β-sheet domain II of Cry1F, CBD 1 & D2 = Carbohydrate-binding domains, EC 2 - ectodomain EC 2).

Based on the Cry1F protoxin being cleaved by midgut digestive enzymes to the activated toxin form and domain II in the activated toxin core being responsible for binding specificity, we first determined the activated Cry1F toxin structure for *in silico* protein-protein interaction (PPI) studies *in vitro* (**Figures S3**, **S4**; **Supplementary file**). None of the selected C-type lectin proteins contained transmembrane regions, while CD63 had four transmembrane regions and two extracellular domains (**Figures S5**, **S6**; **Supplementary file**). The carbohydrate-binding domains of C-type lectins (MMR1, C-MR2, CD209, and C-lectoxin), LBP and the longest extracellular domain in CD63 (EC 2) were used for *in silico* prediction of binding to domain II of Cry1F (IPR001178) (**Figure 5A**). Interactions between these proteins and Cry1F were successfully predicted as PPI models (**Figure 5B**). The HADDOCK Score and root mean square deviation (RMSD) of the protein complexes were assessed using the top first cluster (**Table 2**). The Cry1F-MMR1 PPI had the highest HADDOCK score (-84.1 ± 7.6), while Cry1F interacting with the CD63 E2 domain had the lowest (-29.7 ± 4.8). Overall, the interaction parameters of Cry1F domain II with the tested hemocyte proteins and obtained HADDOCK scores were shown in **Table 2**.

**Table 2:** PPI and clustering scores of Cry1F homology model in complex with C-type lectins and tetraspanin proteins.

Family (Pfam)			Hemocyte protein (s)	Protein domain (s)	Cry1F central β-sheet domain		
					Cluster number/Size^a^	HADDOCK score^b^	Overall RMSD (Å)^c^
C-type lectin (PF00059.21)			MMR1	D1	10/7	-84.1 ± 7.6	0.6 ± 0.5
			C-MR2	D1	6/8	-40.7 ± 5.6	0.1 ± 0.0
				D2	5/7	-73.6 ± 8.9	0.1 ± 0.1
			CD209	D1	11/5	-71.5 ± 15.0	4.1 ± 0.1
				D2	2/18	-42.2 ± 0.5	21.5 ± 0.0
			C-lectoxin	D	1/56	-76.8 ± 1.9	0.1 ± 0.1
			LBP	D1	1/20	-58.4 ± 10.3	13.4 ± 1.1
				D2	4/12	-44.6 ± 4.1	20.7 ± 0.0
Tetraspanin (PF00335.20)			CD63	EC 2	1/30	-29.7 ± 4.8	11.3 ± 0.1

^a)^Cluster size based on a total of 200 generated models, ^b)^scores obtained based on the lowest energy score, calculated as the sum of Van der Waals, electrostatic, desolvation, and restraint energies, as an average of the four best structures of the cluster. HADDOCK scores are expressed in kJ/mol. ^c)^RMSD among the best four structures of the cluster number.

C-type lectins (MMR1, C-MR2, CD209, C-lectoxin and LBP) and tetraspanin (CD63) were successfully expressed and purified in a heterologous system (**Figure S7**; **Supplementary file**) for *in vitro* PPI by surface plasmon resonance (SPR) analysis. Results from SPR experiments supported interactions between activated Cry1F toxin and MMR1, C-MR2, C-lectoxin, LBP or CD63 (**Table 3**). Multiple analyte injections and corresponding association and disassociation curves (**Figure S8**; **Supplementary file**) were used to estimate binding parameters. No interaction was detected between Cry1F and CD209 (**Figure S9A**; **Supplementary file**), and this protein was excluded from further experimentation. The lowest equilibrium disassociation constant (22 nM) was observed for Cry1F-C-MR2 interactions, whereas Cry1F-CD63 had the highest equilibrium disassociation constant (294.4 nM) (**Table 3**). In contrast, MMR1 showed very low response units, preventing estimation of binding parameters.

**Table 3:** SPR evaluation of Cry1F binding affinity with ACB C-type lectins and tetraspanin proteins.

ImmobilizedProtein	Immobilized level (RU)^a^	Analytes	AnalyteConcentration(nM)	Binding parameters		
				K_a_ (M^-1^s^-1^)^b^	K_d_ (s^-1^)^c^	K_D_ (nM)^d^
Activated Cry1F	1763.7	MMR1	3.906-2000	Low response unit		
	1742.2	C-MR2	7.813-500	1.85 × 10^5^	4.10 × 10^-3^	22.1
	1757.5	C-lectoxin	3.906-500	1.10 × 10^5^	5.13 × 10^-3^	46.6
	1742.2	LBP	3.906-2000	2.47 × 10^4^	2.94 × 10^-3^	119.0
	1757.5	CD63	7.813-2000	1.61 × 10^4^	4.74 × 10^-3^	294.4

^a)^RU – Response unit, ^b)^K_a_ - association constant, ^c)^K_d_ - dissociation constant, ^d)^K_D_ - equilibrium disassociation constant.

**Figure 6:**
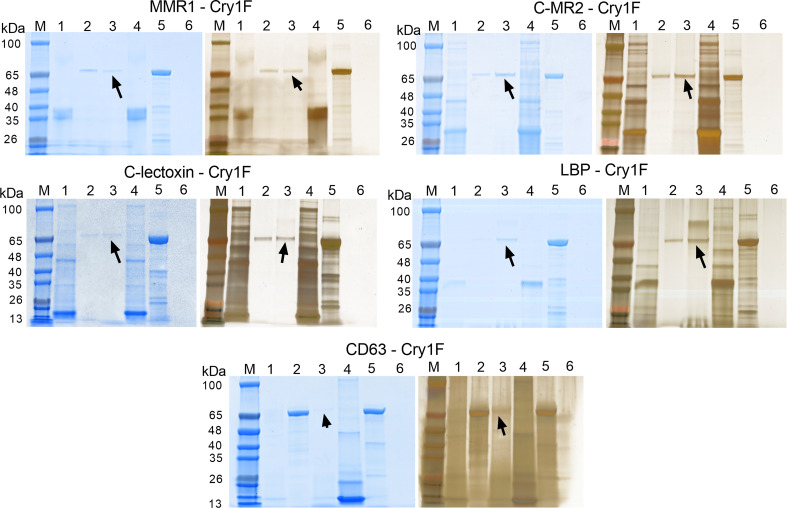
Pull-down assays testing protein-protein interactions (PPI) between Cry1F, C-type lectins and tetraspanin. Each panel presents SDS-PAGE gels initially stained with FastBlue Protein stain (left) and further stained using ProteoSilver silver staining kit (right). Lanes M - TrueColor protein marker, Lanes 1 - Bait flowthrough, Lanes 2 - Prey flow through, Lanes 3 - Final elution, Lanes 4 - Purified bait protein, Lanes 5 - Prey protein (activated Cry1F) and Lanes 6 - Non-treated control. The arrow mark indicates detection of Cry1F protein in the elution, supporting interaction with immobilized bait proteins. ACB proteins tested: Macrophage mannose receptor 1-like (MMR1), C-type mannose receptor 2-like (C-MR2), CD209 antigen-like protein E (CD209), C-type lectin lectoxin-Phi1-like (C-lectoxin), hemolymph lipopolysaccharide-binding protein (LBP), and CD63 antigen-like (CD63 - ectodomain EC2).

As a second *in vitro* method to test for PPI, we performed pull-down assays with activated Cry1F as a prey protein and C-type lectins and tetraspanin as immobilized bait proteins (**Figure 6**). Eluted samples contained Cry1F independently of the bait protein used (**Figure 6**, lanes 3), denoting interactions between the toxin and the immobilized hemocyte proteins. Despite the low binding between MMR1 and Cry1F detected in SPR analyses, pull-down assays detected interactions between these proteins (**Figure 6**). In agreement with SPR results, the pull-down assay did not detect interactions between Cry1F and CD209 (**Figure S9B**; **Supplementary file**).

### Activation of MAPK in hemocytes of ACB-BtS and ACB-FR after intoxication with Cry1F toxin

**Figure 7:**
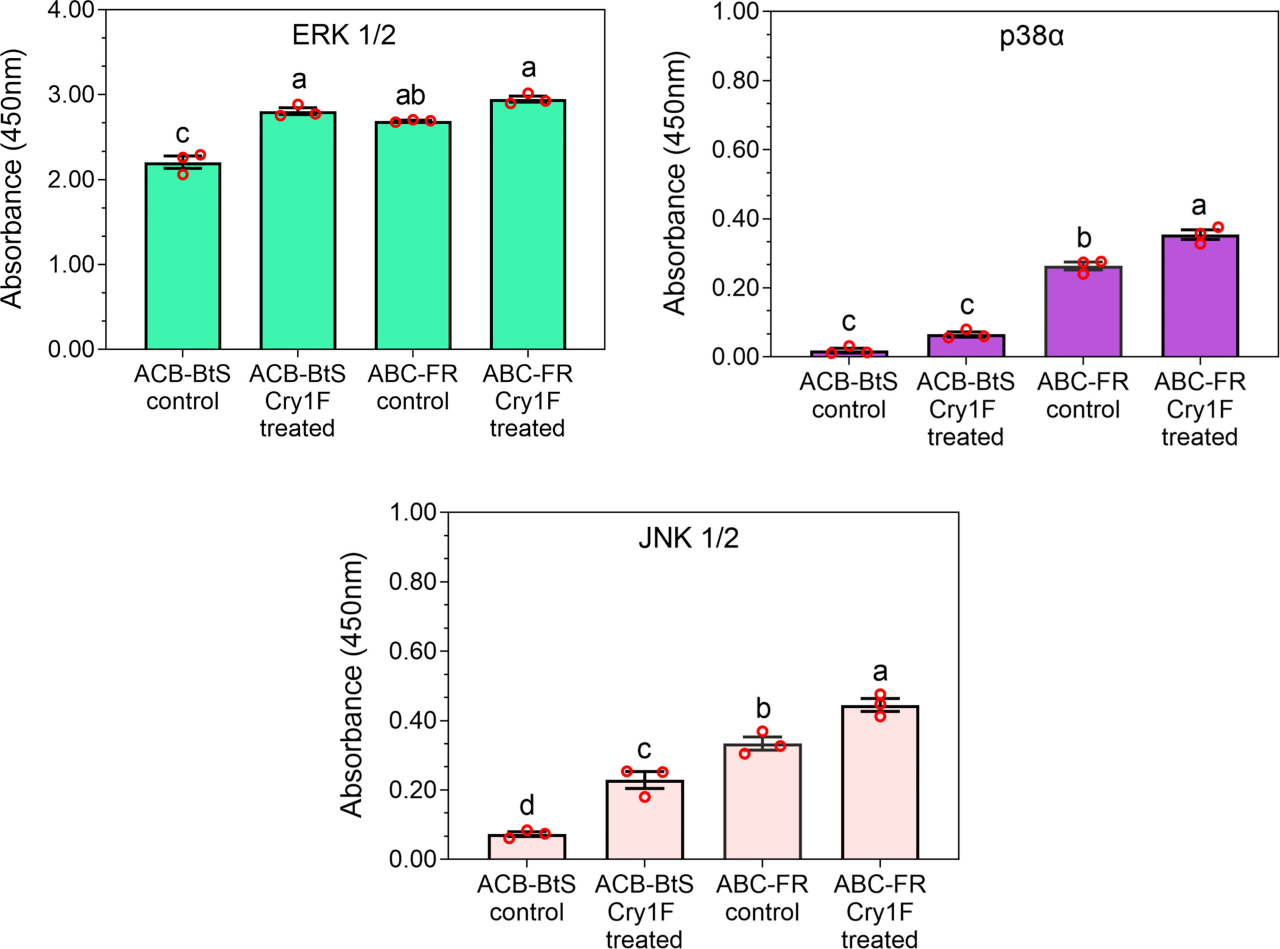
Activity of selected mitogen-activated protein kinases (MAPKs) in hemocytes from ACB-BtS and ACB-FR larvae in response to Cry1F treatment. MAPK activity (mean and corresponding standard errors from three biological replicates) in hemocyte lysates after 48 h of treatment with Cry1F toxin (1,075 μg/g; toxin/diet). Antibody cocktails detected MAPK family kinase enzymes ERK 1/2, p38α, and JNK 1/2. Different letters on vertical bars represent statistically significant differences between treatments for the specific gene tested (One Way ANOVA and *post hoc* LSD Tukey test; *P* < 0.05).

The activity of MAPKs was measured in hemocyte lysates after 48 h of larval treatment with a control or Cry1F diet. Exposure to the Cry1F diet increased ERK1/2, p38α, and JNK1/2 activity in hemocytes from larvae of ACB-BtS and ACB-FR strains (**Figure 7**). In control treatments, activity of tested MAPKs was relatively lower in ACB-BtS compared to ACB-FR hemocytes. In ACB-BtS, the activity of both ERK1/2 and JNK1/2 was significantly increased after Cry1F exposure, while p38α activity was not significantly changed compared to control (**Figure 7**). In contrast, treatment with Cry1F significantly increased p38α and JNK 1/2 activities in hemocytes from ACB-FR, while ERK1/2 activity was not significantly changed (**Figure 7**).

### CD63 tetraspanin gene knockdown and resistance to Cry1F

**Figure 8:**
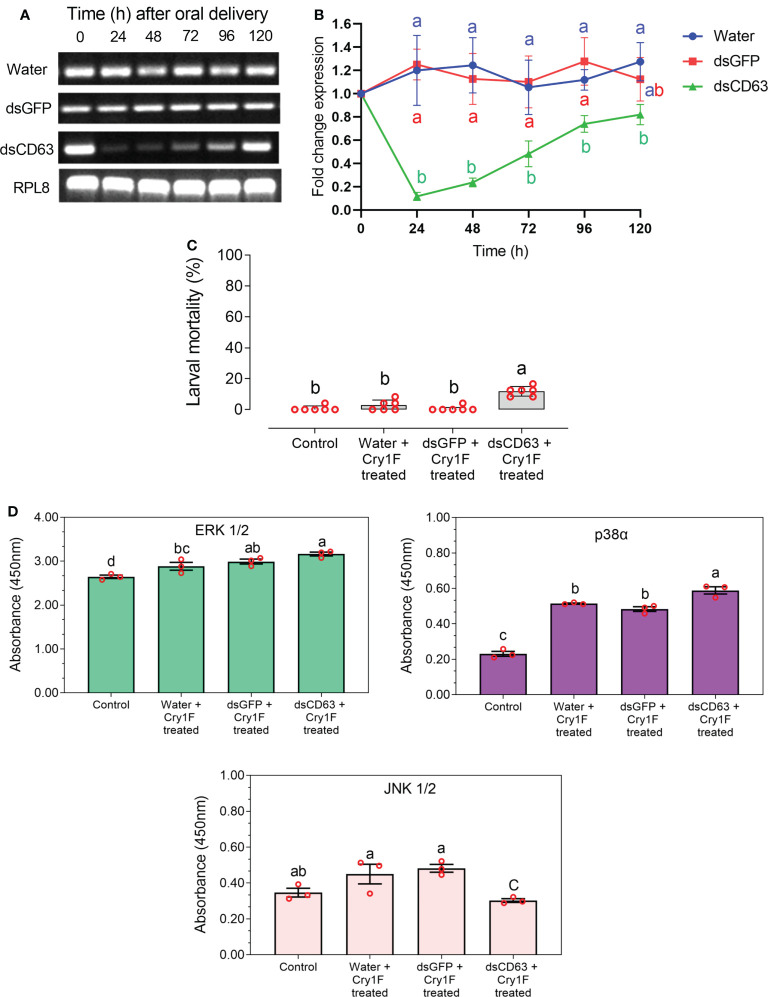
Functional testing of CD63 tetraspanin as putative Cry1F toxin receptor in ACB-FR using silencing by RNAi. **(A)** Amplified PCR product of CD63 gene after RNAi at different time points and compared with internal controls. **(B)** Relative mean fold CD63 expression ( ± SE) from three biological replicates detected using qRT-PCR. Different letters represent significantly different transcript levels (Tukey test, *P*< 0.05). **(C)** Mean percentage mortality after 48 h in ACB-FR larvae exposed to control or diet with Cry1F alone or in the presence of dsRNA targeting GFP or CD63. Shown are means and corresponding SE estimated from six independent bioassays represented by red circles. Different letters represent significant differences (Tukey test, *P*< 0.05). **(D)** MAPK activity in hemocyte lysates after CD63 gene knockdown and treatment with Cry1F (LC_50_ dose) for 48 h in the ABC-FR strain, compared with control group. Blank values were subtracted from the respective experiments. Vertical bars under different letters were statistically significant and same letters were not significant (One Way ANOVA and *post hoc* LSD Tukey test, *P*<0.05).

Based on previous reports of linkage between resistance to Cry toxins and a tetraspanin [20], we chose CD63 for functional tests as a putative Cry1F receptor. Treatment with dsCD63 significantly silenced CD63 gene expression after 24 hours, and the effect lasted until 96 hours (**Figures 8A, B**). In contrast, control treatments with dsGFP or water had no effect on CD63 expression. Bioassays with dsRNA-treated ACB-FR larvae and a sublethal dose of Cry1F detected a significant increase in susceptibility to the toxin in larvae treated with dsCD63 compared to controls (**Figure 8C**).

Hemocytes were collected from larvae treated with dsRNA and used to detect MAPK-ERK 1/2, p38α and JNK 1/2 activity. A significant increase in activity was detected for p38α and ERK 1/2 for all treatments when compared to control (**Figure 8D**). The most drastic difference unique to treatment with dsCD63 and Cry1F was a significant reduction in JNK 1/2 activity levels (**Figure 8D**).

## Discussion

Disruption of the gut epithelial barrier by Cry proteins and subsequent collapse of the gut epithelium allows passage of Bt and gut microbiota into the hemocoel, onsetting lethal septicemia (42). Microbes (including Bt) entering the hemocoel meet hemocytes circulating in the hemolymph, triggering the host’s innate immune response. In this work we tested for interactions between circulating hemocytes and hemolymph enzymes with Cry1F, including larvae from susceptible and Cry1F-resistant ACB strains. Transcriptomic and proteomic analyses of hemocytes after Cry1F treatment detected more genes with increased expression in ACB-FR than ACB-BtS larvae, particularly genes involved in detoxification and immunity.

A common observation in in response to Cry1F in both strains was an increase in GST expression, a protein involved in defense and tissue protection against xenobiotics and insecticides (43). Increased antioxidant enzyme and GST activities were detected in *H. armigera* larvae fed Cry1Ac protoxin (44) and *Galleria melonella* exposed to *B. thuringiensis* ssp. *Galleriae* (45). Midgut tissue damage by Cry3A toxin in *Leptinotarsa decemlineata* triggered elevated levels of reactive oxygen species and a corresponding detoxification response including increased GST expression (46). Interestingly, increased GST activity in response to Cry1F was significantly higher in ACB-FR compared to ACB-BtS, suggesting it may contribute to the resistant phenotype. In agreement with this observation, Cry1F-resistant *Spodoptera frugiperda* (47) and Cry1Ab-resistant *Diatraea saccharalis* (48) displayed constitutively increased GST gene expression or activity when compared to susceptible larvae. In *S. frugiperda* this increased GST activity also associated with reduced susceptibility to tested pesticides. In contrast, feeding on transgenic corn producing the Cry1Ab protein resulted in reduced GST activity in *O. furnacalis* (49). One potential explanation for this discrepancy may be the different effects of feeding on an artificial diet versus corn tissue on GST expression, independently of exposure to Cry proteins.

Immune genes are relevant in the response to Bt intoxication. Hemocytes from *H*. *virescens* larvae that were fed Cry1Ac toxin showed higher expression of immune genes, such as antimicrobial peptides, cytokines and protease inhibitors (50). Elevated secretion of antimicrobial factors was observed in *Galleria melonella* exposed to *B. thuringiensis* ssp. *Galleriae* (51). Rahman et al. (52) reported an elevated immune status detected as increased hemolymph melanization associated with resistance to Bt in larvae of *Ephestia kuehniella.* Transcriptomic and proteomic comparisons identified 5 immune genes displaying altered expression after treatment with Cry1F in ACB-FR but not in ACB-BtS: MMR1, C-MR2, beta-1 3-glucan-binding-like, lysozyme-like and phenoloxidase-activating factor 2-like proteins. The phenoloxidase-activating factor 2-like protein activates the prophenoloxidase cascade following the recognition of pathogen-derived products (53). The resulting increased phenoloxidase activity results in higher hemolymph melanization, as detected in *Galleria melonella* exposed to *B. thuringiensis* ssp. *Galleriae* (54). Importantly, Cry1A-resistant strains of *O. furnacalis* (31), *H. armigera* (55) and *S. frugiperda* (56) showed higher levels of hemolymph phenoloxidase activity when exposed to Cry1A toxins compared to susceptible insects, suggestive of a potential contribution to the resistant phenotypes.

Based on common detection in transcriptomic and proteomic analyses, we selected five C-type lectins (MMR1, C-MR2, CD209, C-lectoxin and LBP) and a CD63 tetraspanin that were differentially enriched in ACB-FR to test interactions with Cry1F toxin. The C-type lectin family includes pattern-recognition proteins (PRPs) that bind carbohydrate moieties in pathogens to activate the innate immune response (57–59). Using RACE-PCR we detected MMR1, C-MR2, CD63, and LBP expression in midgut tissue (**Figure S10**; **Table S2**; **Supplementary file**), supporting additional opportunities for interaction with Cry1F toxin. The CD63 tetraspanin is involved in cell adhesion, activation, differentiation and tumor invasion (60).

Protein docking models showed good interaction between the Cry1F domain determining binding specificity (61) and the selected hemocyte proteins. Results from *in vitro* PPI testing also supported that C-MR2, C-lectoxin, LBP and the CD63 tetraspanin bound Cry1F, while interactions with MMR1 were relatively weak and CD209 does not interact with Cry1F. Binding of hemocyte C-type lectins and tetraspanin to Cry1F may reflect a defensive step in the immune system responding to a bacterial toxin challenge. These interactions could trigger activation of the cellular immune response, as suggested by DEGs and DAPs detected in our RNA-Seq and iTRAQ analyses. Hemocyte-mediated immune mechanisms could help neutralize Cry1F toxicity in the hemolymph.

Interaction with CD63 activates downstream signaling of the MAPK pathway (62), which is known to be involved in defense against and resistance to Cry toxins (18). Under control conditions, all tested MAPKs (ERK1/2, p38α, and JNK1/2) displayed significantly higher activity in ACB-FR compared to ACB-BtS. This observation suggests that larvae from the ACB-FR strain are in an elevated immune status, as previously described for Bt-tolerant *E. kuehniella* (52). Treatment with Cry1F increased hemocyte kinase activity in larvae from both ACB-BtS and ACB-FR strains Knockdown of CD63 in hemocytes of ACB-FR larvae resulted in increased susceptibility to Cry1F, supporting that CD63 tetraspanin contributes to resistance against Cry1F. Knockdown of CD63 expression also significantly affected the levels of MAPK activity in ACB-FR hemocytes, with JNK 1/2 activity levels uniquely reduced in knockdown hemocytes compared to treatments with Cry1F alone or with dsGFP. This observation suggests an interplay between CD63 and the JNK 1/2 p athway, one of the major known immune pathways participating in defense against Bt infection (63–67) and Cry intoxication (68, 69). Although speculative, a CD63 (tetraspanin)-JNK interplay may help explain why a mutation in a tetraspanin gene would result in increased Cry1Ac binding and at the same time resistance against that toxin in *H. armigera* (21). It is plausible that the tetraspanin mutation increased binding affinity for Cry1Ac and thus facilitated immune activation compared to susceptible insects. In a distinct approach to potentially increase tetraspanin-Cry toxin interactions and thus enhance the defensive response, three tetraspanin genes were up-regulated in Cry1Ac-resistant compared to susceptible *Helicoverpa zea* (69).

Overall, t his study identifie candidate hemocyte proteins that may be involved in activating cellular defensive responses to Cry intoxication. Our findings suggest that Cry toxins can interact with immune receptors, some expressed in gut cells and in hemocytes, to potentially activate a defensive response. After Cry toxins disrupt the epithelial barrier, an activated immune response may be critical against gut microbiota entering the hemocoel, increasing the chances of survival. Further work is needed to characterize the role of identified hemocyte proteins in the molecular response to Cry intoxication and their potential contribution to resistance in ACB-FR. In addition, the current analysis focused on up-regulated immune-related genes, but down-regulated genes and potential mutations may also participate in resistance in the ACB-FR strain.

## Conclusion

Results from this study support potential participation of C-type lectins and a tetraspanin in the defensive response to Cry intoxication and contribution to the resistance phenotype in ACB-FR. Up-regulation of the immune response would be critical to defense against gut microbiota entering the hemocoel after Cry intoxication. Interactions of Cry1F toxin with the CD63 antigen receptor may activate the MAPK-JNK 1/2 pathways in hemocytes to mount a cellular response to intoxication. Genetic knockdown of CD63 results in increased susceptibility of ACB-FR larvae, suggesting participation of this gene in the resistant phenotype.

## Data availability statement

The datasets presented in this study can be found in online repositories. The names of the repository/repositories and accession number(s) can be found below: https://www.ncbi.nlm.nih.gov/, PRJNA723187.

## Author contributions

Conceptualization: KLH. Experiment design: SP, DPJ and KLH. Investigation: SP and DPJ. Formal analysis: SP, DPJ, ZYW and JLJ-F. Funding acquisition and resources: ZYW. Writing - original draft: SP and DPJ. Writing - review and editing: KLH, ZYW and JLJ-F. All authors contributed to the article and approved the submitted version.

## Funding

The work was supported by the Agricultural Science and Technology Innovation Program (ASTIP) from the Chinese Academy of Agricultural Sciences (CAAS).

## Acknowledgments

JLJ-F acknowledges Hatch Multistate Research Project NC-246 from the US Department of Agriculture National Institute of Food and Agriculture support for this study.

## Conflict of interest

The authors declare that the research was conducted in the absence of any commercial or financial relationships that could be construed as a potential conflict of interest.

## Publisher’s note

All claims expressed in this article are solely those of the authors and do not necessarily represent those of their affiliated organizations, or those of the publisher, the editors and the reviewers. Any product that may be evaluated in this article, or claim that may be made by its manufacturer, is not guaranteed or endorsed by the publisher.
