# Supplementary material for: Hemocyte response to treatment of susceptible and resistant Asian corn borer (Ostrinia furnacalis) larvae with Cry1F toxin from Bacillus thuringiensis
Source: Front Immunol. 2022 Nov 17;13:1022445. doi: 10.3389/fimmu.2022.1022445 (PMC9714555; doi:10.3389/fimmu.2022.1022445)
Supplement: Supplementary file 1 [file DataSheet_1.docx]

**Supporting Information**

**Gene validation from hemocyte total RNA**

PCR reactions were performed using GoTaq® Green Master Mix (Promega, USA) following the manufacturer’s protocol. The amplification program was set as follows: Initial denaturation for 5 minutes at 94 °C; 36 cycles of 1) 94 °C for 30 s; 2) 60 °C for 30 s; 3) 72 °C for 30 s; and a final extension for 10 minutes at 72 °C. The target and reference genes selected for the study were amplified and validated by gene sequencing. The sequenced PCR products were checked in NCBI BLAST, and accession numbers were retrieved (Table S1).

**Identification of Cry1F peptides**

The activated Cry1F toxin was added in 10 kDa Amicon® ultrafiltration tube (Merck, Darmstadt, Germany) and treated with dithiothreitol (1 mM, pH 7.0) in a 1:40 ratio. To ensure protein reduction, the mixture was vortexed and placed in a water bath at 37°C for 1 hour. The protein in the ultrafiltration tube was resuspended with 300 μL iodoacetamide (1 M) and incubated at RT in the dark for 30 minutes to ensure alkylation. The sample in an ultrafiltration tube was washed thrice using 300 μL triethylammonium bicarbonate buffer (TEAB, 0.5M, pH 8.5) by centrifugation (13, 400 × *g*, 10 min, 4°C). The solution collected in the collection tube was discarded. The filter interface was placed on a new collection tube, 50 μL (0.5 μg/μL) of sequencing grade modified trypsin (Promega, WI, United States) was added and incubated at 37°C overnight. After trypsin digestion, 100 μL of TEAB buffer was added to the filter interface containing peptides and centrifuged (13, 400 × *g*, 10 min, 4°C), and this process was repeated thrice. The digested peptides were collected in a collection tube and desalted using Ziptip^®^ C18 solid-phase extraction tips (Merck, Darmstadt, Germany). Initially, the Ziptips were activated by pipetting (3 times) in acetonitrile (100 %) and equilibrated by pipetting (5 times) in acetonitrile (2%) and followed by formic acid (0.1%). Further, the sample in the collection tube was aspirated repeatedly (10 times) to ensure the bonding of digested peptides inside the Ziptip^®^ C18 tips. The bonded peptides in the tip were desalted by pipetting (5 times) in acetonitrile (2%) and formic acid (0.1%). Finally, the sample from Ziptip was eluted in 10 μL of acetonitrile (50%) and followed by 10 μL formic acid (0.1%) by repeatedly pipetting (10 times). The eluates in acetonitrile (50%) and formic acid (0.1%) were mixed together. The collected eluent was vacuum dried and stored at -80°C until further analysis. Dried polypeptide samples were dissolved using 20 μL of 0.1% formic acid and loaded in an EASY-nLC™ 1200 System (Thermofisher Scientific, MA, USA). The Easy-nLC liquid phase was separated by low pH reversed-phase C18 capillary chromatography (150 μm 150 mm, 1.9 μm) with 99.9% H_2_O and 0.1% formic acid as A phase and 80% acetonitrile, 19.9% H2O and 0.1% formic acid as B phase. The effective elution gradient was 6-35%, the total elution time was 60 min, and the flow rate was 0.5 μL/min. Orbitrap Fusion™ mass spectrometer (Thermofisher Scientific, MA, USA) was used to analyze and identify polypeptide mixtures in a high-sensitivity mode.

Instrument parameters were set to high-speed signal-dependent scan, scanning time was 60 min, the first-level full scan was set to 60,000 resolutions, scanning range was 300-1500 m/z, and the maximum injection time was 100 millisecond. After each first-level scan, secondary scan resolution was set to 15,000 with 32% collision energy. The mass spectral data obtained were retrieved by Mascot v2.6.0. A peptide mass tolerance of 10 ppm and fragment mass tolerance of 0.5 Da were acceptable. Spectra were analyzed with Scaffold Q+ v4.6.2 (Proteome Software Inc., Portland, OR, USA) for quality with >99% protein identification probability and FDR <1%.

**Surface plasmon resonance analysis (SPR)**

The macromolecule interactions between hemocyte proteins MMR1, C-MR2, C-lectoxin, CD63 or LBP with Cry1F were analyzed on Biacore™ 8K.

**Immune-related genes localization in midgut tissue**

Midgut tissue was dissected from the ACB-BtS and ACB-AhR strains. About 5 larvae were used for midgut extraction. The midgut tissue was excised from the foregut and hindgut, and a longitudinal slit was made in the midgut tissue to remove the food and other debris. Dissected midgut tissue was rinsed with ice-cold Mannitol buffer (300 mM Mannitol, 17 mM Tris-HCl, pH 7.2) and the tissue was picked with fine forceps and put on filter paper to remove the excess liquid. Total RNA was extracted from hemocytes and midgut using TRIzol^®^ (Ambion, Life Technologies, CA, USA), following the manufacturer's instructions. The RNA concentration and quality were evaluated using NanoDrop 2000 spectrophotometer (NanoDrop products, USA).

The selected MMR1, C-MR2, LBP and CD63 genes Open Reading Frame (ORF) regions were analyzed using DNASTAR Lasergene v 17.3, and primers were designed (Table S2). C-lectoxin gene was not detected in midgut tissue. Rapid amplification of cDNA ends (RACE) technique was performed to find the full-length sequence of selected genes open reading frames (ORFs) from the cDNA synthesized from hemocyte (template) or midgut tissue (template) using 5’- and 3’ - RACE Kit (Tiandz, Inc., China). The RACE-PCR conditions, initial denaturation at 95°C for 5 min followed by 35 cycles of 95°C for 30 s, 57°C for 30s, 72°C for 3 min, followed by a final extension of 10 min at 72°C. The PCR products were analyzed using 1% agarose gel electrophoresis, and the bands were excised, purified and sent to sequencing. The gene sequences of MMR1, C-MR2, LBP and CD63 were translated to protein sequence using DNASTAR Lasergene v 17.3. The protein sequences of hemocyte and midgut were aligned using ESPript v 3.0 (<https://espript.ibcp.fr/ESPript/ESPript/>)

**Supplementary Tables and Figures**

**Table S1** Primer sequences used for the study.

| **Primer** | **Target gene** | **Primer sequence (5’ – 3’)** | **NCBI Acc. No.** |
| --- | --- | --- | --- |
| 1 | Glutathione S-transferase 1-like | FP - TCTAACGCCCGTATTCACAA  RP - ACGCACGTATTCACAAACGA | XM_028318786 |
| 2 | Cytochrome P450 4C1-like | FP - ACTCCTTGGCATCCATCAAG  RP - AGTGTAGAGCCGCATGCTTT | XM_028312907 |
| 3 | Cytochrome P450 306a1 | FP - TACCCGCCTGAAGACAAAAC  RP - TCCACGTACCAACACCTCAA | XM_028316836 |
| 4 | UDP-glucuronosyltransferase 2B15-like | FP - ACCTTCGAGCCTCTCCTAGC  RP - GGAATCCACCGACAAACCTA | XM_028310567 |
| 5 | C-type mannose receptor 2-like | FP - CATCTGACATCGTCGGTTTG  RP - ACTGTTGATGATGGCCAGGT | XM_028308123 |
| 6 | CD209 antigen-like protein E variant X3 | FP - CAACAGATGCCCCTAAGGAA  RP - GTCTCCACCATCAGGTTGCT | XM_028314999.1 |
| 7 | Toll-like receptor 2 | FP - GGCTACCTCTGGCTCCTTCT  RP - CACCCGGAGTATCGGTCTAC | XM_028315968 |
| 8 | Hemolymph lipopolysaccharide-binding protein-like transcript variant X1 | FP - CTACAAAATTCCACGCGACA  RP - GAAGGAAGTGCGGAGGTATG | XM_028302111 |
| 9 | Beta-1,3-glucan-binding protein-like | FP - AAGCTGGGCGACAAAGTCTA  RP - GCGTTGTTGTAGGGTCAGGT | XM_028323618 |
| 10 | Phenoloxidase-activating enzyme-like | FP - ACCAGCTACAGCGAGGAGAA  RP - AATTCGCACGTCCAAATCTC | XM_028313261 |
| 11 | Caspase 1 like variant X1 | FP - CACACGTCGAGTTCCTTCAA  RP - GGTGGTTCTCGTCAGCATCT | XM_028322910.1 |
| 12 | Lysozyme-like | FP- CCCTTCGCTTACGTCAATGT  RP - CTCCAAAGTTGCCAAATGCT | XM_028305654 |
| 13 | Ribosomal protein L8  (Reference gene, Hemocyte) | FP - ACTGCGGAAAGAAGGCTACA  RP - ACGTTTGGCATCAGGATTGT | XM_028306632.1 |
| 14 | dsCD63 | FP -GGATCCTAATACGACTCACTATAGGCATCATTATACTCGCCGTTGGC  RP - GGATCCTAATACGACTCACTATAGGCTATGCCTATAGAACCAGCATG | XM_028322859 |
| 15 | GFP | FP-GGATCCTAATACGACTCACTATAGGGTGGTCCCAATTCTCGTGGAAC  RP- GGATCCTAATACGACTCACTATAGGGCTTGAAGTTGACCTTGATGCC | AB062168.1 |

**Figure S1.** PCR amplification of selected genes before qRT-PCR analysis. Lane M - Transgene 2K plus marker. 1. Caspase-1-like variant X1, 2. Lysozyme-like, 3. UDP-glucuronosyltransferase 2B15, 4. Cytochrome P450 4C1-like, 5. Cytochrome P450 306a variant X2, 6. CD209 antigen-like protein (endocytosis), 7. Glutathione S-transferase 1-like, 8. LPS lipopolysaccharide-binding protein-like, 9. C-type mannose receptor 2-like, 10. Phenoloxidase-activating enzyme-like, 11. Toll-like receptor 2, 12. Beta-1 3-glucan-binding protein-like.


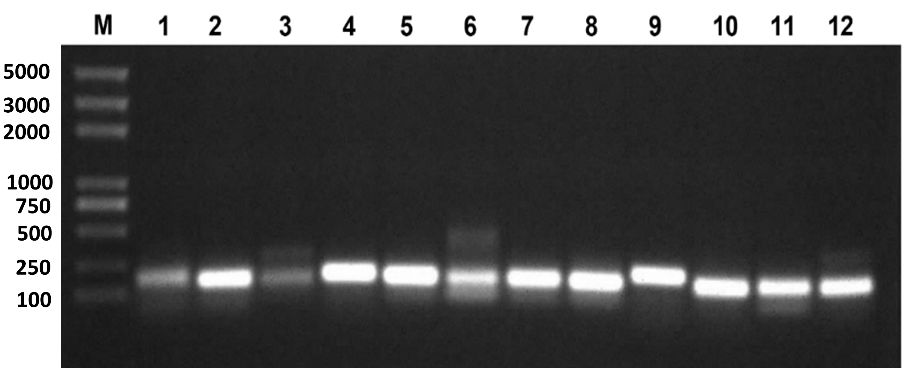


**Figure S2.** Differentially expressed gene count accounted for larvae from the ACB-FR (resistant) strain treated (RT) vs. control (RC) and susceptible ACB-BtS treated (ST) vs. control (SC).


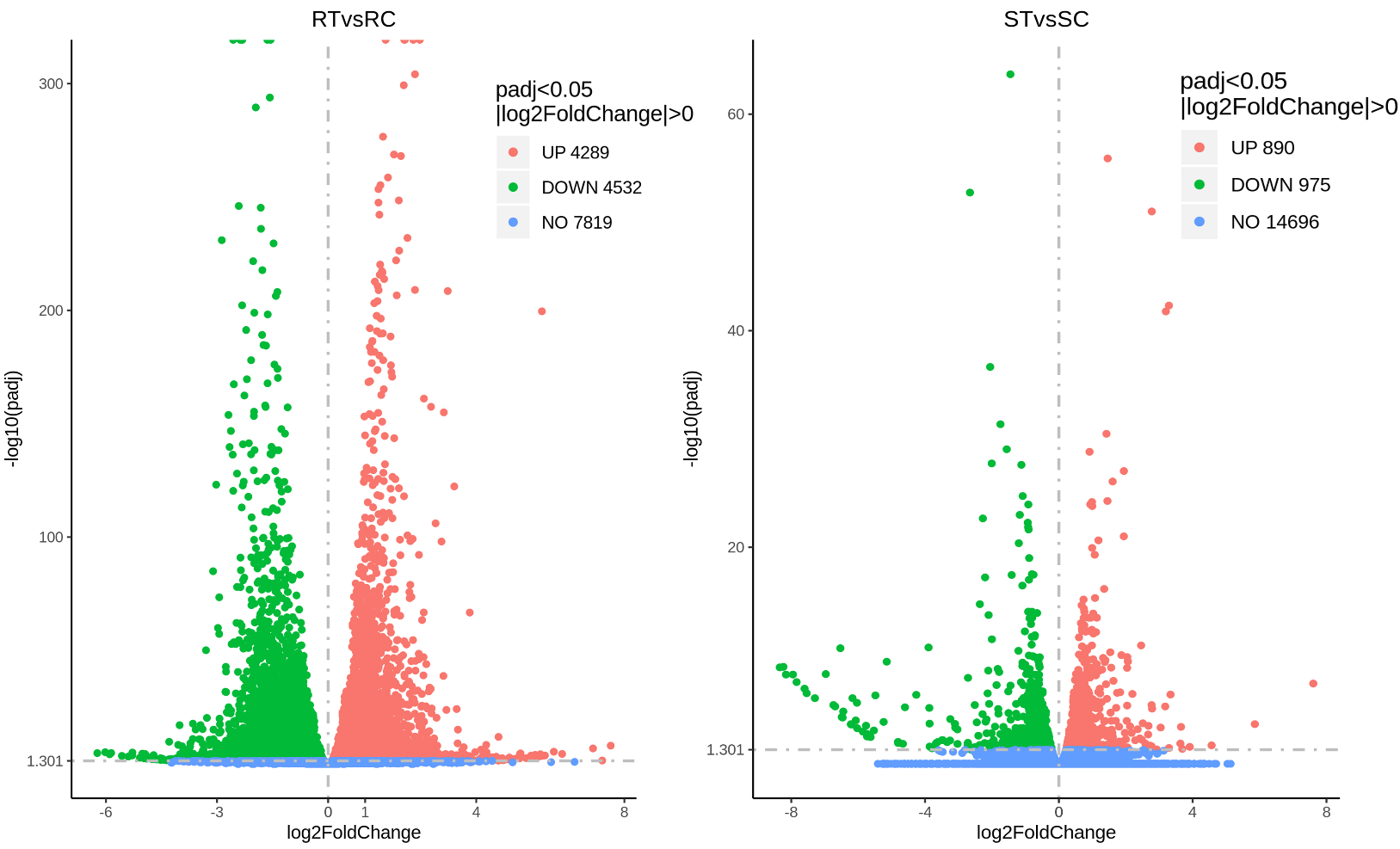


**Figure S3.** Coverage of Cry1F sequence in proteomic LC-MS/MS analysis of Cry1F toxin. Detected peptides are highlighted in yellow.

**Figure S4.** Prediction of Cry1F toxin domains for protein-protein interaction studies. A) Predicted Cry1F activated region from *in silico* digestion of Cry1F protoxin with Expasy PeptideCutter. Amino acid sequences boxed in green represent the N-terminal helical bundle of domain I, the blue color box represents the central beta-sheet of domain II, and pink colors represent the C-terminal beta-sandwich of domain III. The amino acid sequences marked with red color represent the regions mapped by LC-MS/MS partial analysis in this study. B) Structure homology model of Cry1F presenting the three domains. C) SDS-PAGE gel (12 %) of purified Cry1F toxin used for the LC-MS/MS analysis. Lane 1 - Precision Plus Protein Dual Color Standards (Bio-Rad, CA, USA), lane 2 - Activated Cry1F protein.


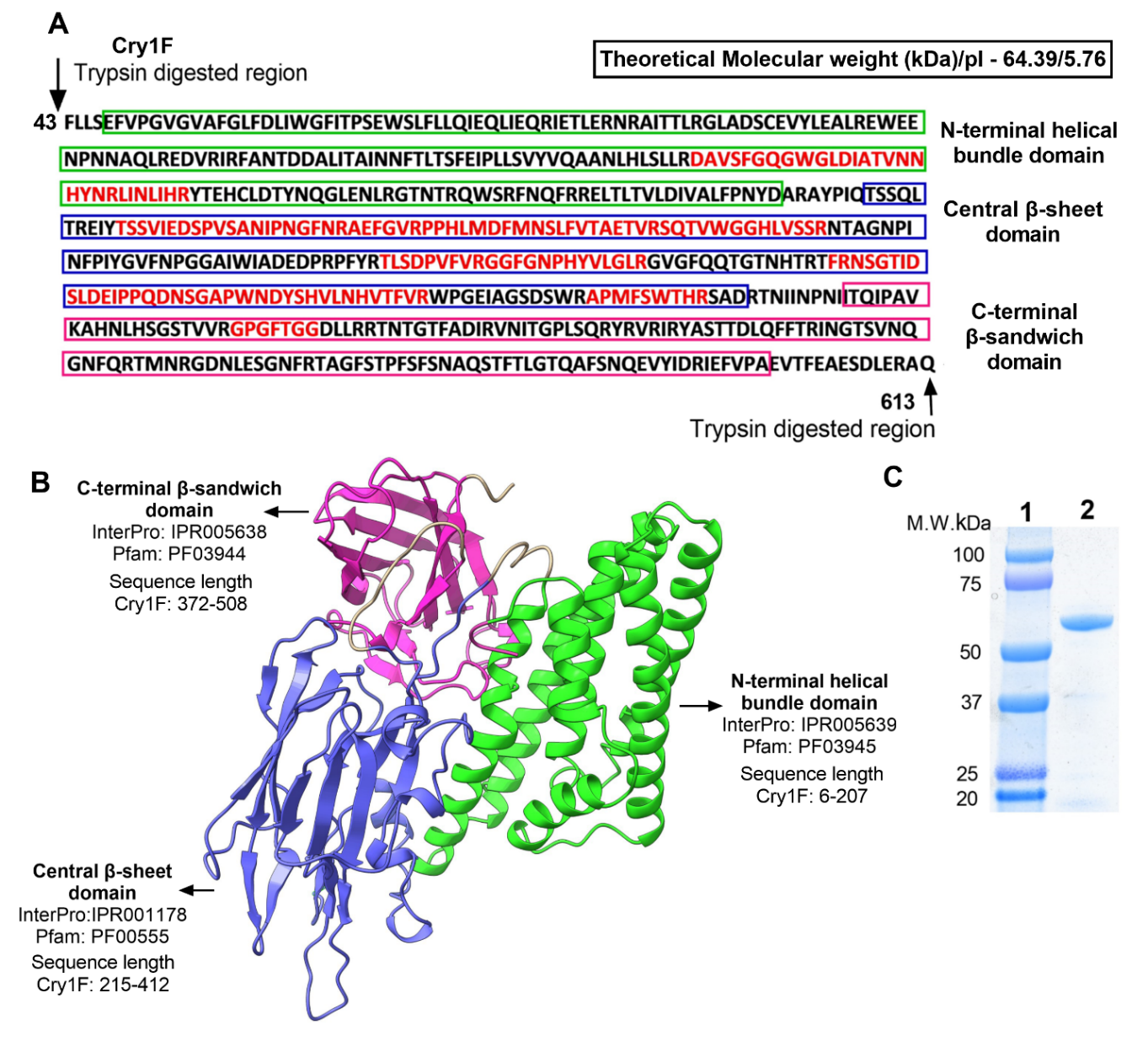


**Figure S5.** C-type lectin and CD63 tetraspanin sequences

**MMR1 - 36.6 kDa, pI=5.12** (NCBI Acc. No. XP_028170786)

QREKKFFRKDYKYIEATESFYKIHTIHKTWQDAKDVCAMEGATFFVPEDQGEADAVLAFWNATQPYSWVYIGVSSLIVKGVFETVEGQPISDVYSNWGPGEPNDANGEEDCVILRRDGTLNDVNCVNKYPFVCKKTLDSLEWNIKCDMPNMDYVFNEALGKCYKFHLDPRTWREAYAVCSAESSYLAIIDSQAEADHLVKITAEAPKDDVQGDFLRGAVHLGFHKRKDGWKTIRGTKLENSGYSKWGNQQPDGGDNETCGTMFYNGHLNDLKCDHKCFFICEHDVGSLSNSLDFKFGDDE

**C-MR2 - MW=34.6 kDa, pI=6.93** (NCBI Acc. No. XP_028163924)

SELNTSKSNWFRPDYSYSERTGGWFKFHSVPATWEDARLQCYYEGAELVSPFNENIIQKMIMLMDVNEPYIFTGIHSTFSKGVYTSVGGVPLHEMPVSLHSRDVSGNCVTMRSNGRVEARNCSSQYPYICFKKGPEHLTSSVCGADQEYKYEQRTGSCYKFHTLGRTWPQAFRVCVAEGGHLAIINSDVEADVIRGIFQNYPDDAFKADAKYAASIGFQGWGAVKIWWTIHGQTLQDAGYSKWDKFVPDMRSTRHYCGAVGRNGTLSHIECEGVTFPAICEKKADLV

**CD209** - **MW=36.0 kDa, pI=4.73** (NCBI Acc. No. XP_028170800)

QRDNKYFRKDYTYIEATDSFYKMHTIHKTWENAKDLCAMEGATLFYPENQEEADAVSLYWNATQPFPHIFIGASSLIAKGIFETVDGQLISDVYSNWAPGEPNDSSGNEDCVSMNKDQTLNDLSCEHKSPFVCKKTLDSLDWNTKCDIPYLDYEFDETLGKCYKFYSEPRTWRDAYVACSAELSYLAIIDSQAEADHLAKITTDAPKEDSQNALEDTVFLGFNKLRKGWKTIKGTKLQNSGYTKWGDQQPDGGDSESCGGMSYDGLLKDAKCEDQSYFICEHDVATSYNLHERFGD

**C-lectoxin** - **MW=19.3 kDa, pI=5.87** (NCBI Acc. No. XP_028165510)

DVPKDYIVNPNDSHGYKLMYHAKTWTEARDDCANVGAKLAVPKTRDQFEFIQKIVRSMQYQSIVGTEYKLLVWLGISNLKNYQVWANVDGENIEDTEFHTWAGQNGLKSENPAEPHCVGMDSMNFGLRDWWCHQRQPYICEILTVQNATQ

**LBP - MW=34.5 kDa, pI=5.46** (NCBI Acc. No. XP_028157912)

QKRYRNDYVYDANTDAFYKFHVNMAVHWRALTICKLEGATLMVPTSVQQIAQVHGMFKQYPDIGHYVWIGDDNADHESAEETPIIDLQPEVETEAPWERRCLVLTRRGEIQTQNCRYYQSFVCMVEAKNAPYDHRCDVYAAGYEYNTDVKSCYKIPRDIKTWNAAYAECQAHGAHLVVVNSVTERDVLQALMSRTPYLRTSFSPYHYIAGIRATSVIGSEVVYRTIFNETLEEAGFSEWADNEPNNQQGIEYCGSLHQRSGKYNDISCLSSFGYICEKERNE

**CD63 - MW=12.6 kDa, pI=5.27** CD63 (EC2 domain; NCBI Acc. No. XP_028178660).

KGNAEALVQKQLYETMQLYTHDVVVTRLWDEVQEDFSCCGVNNASDWLAPLGTANTDQGLPLSCCQFPFGTITVFNCTMATPAPTLRTEGCSTAFGAWVQSHAGS

**Figure S6.** Topology prediction for selected immune protein sequences by TMHMM Server v2.0. Lectin and ectodomain were used for protein expression.

**
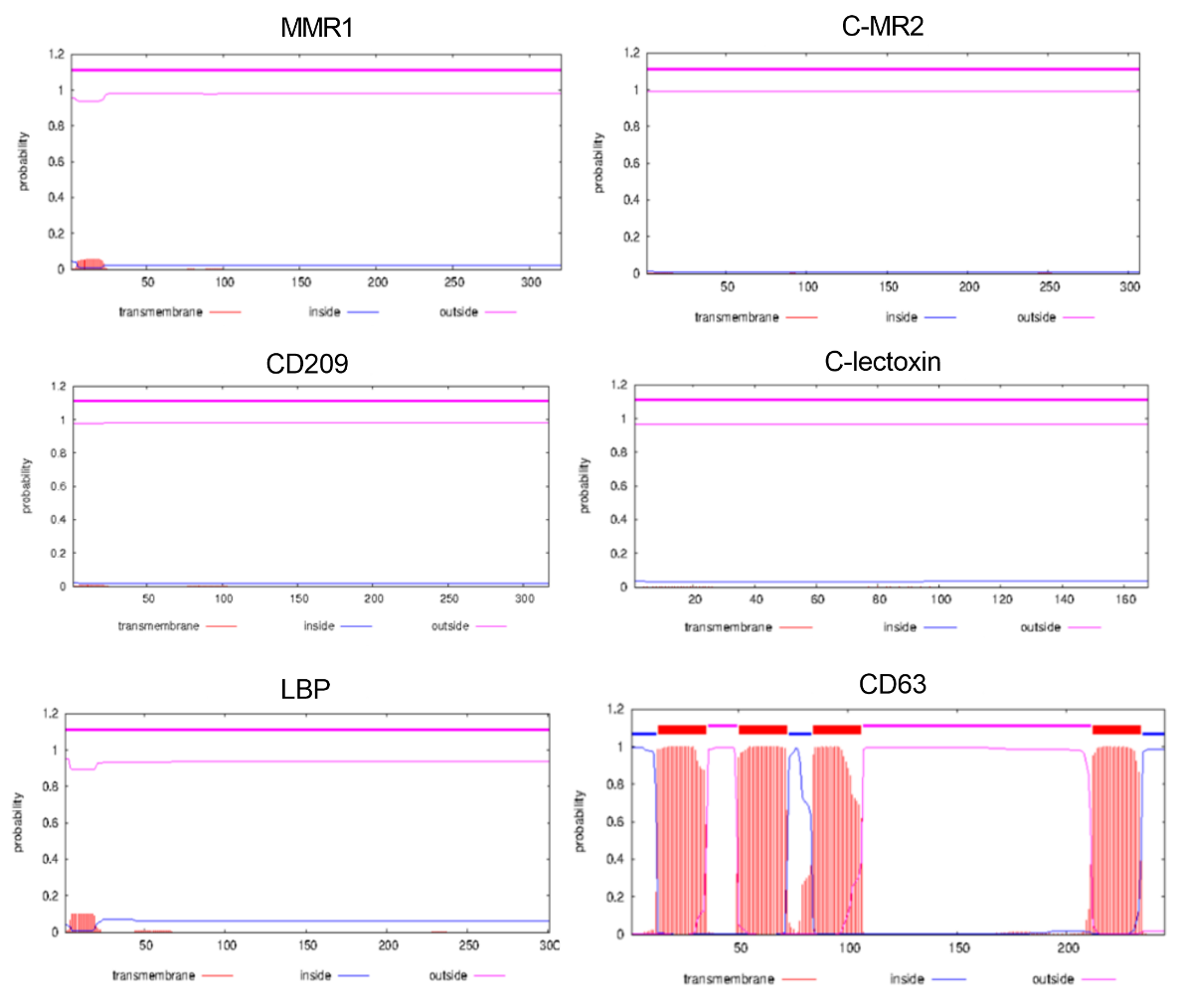
**

**Figure S7.** Hemocyte proteins expressed in a prokaryote system and analyzed in (A) 12% SDS-PAGE and (B)Western blot. MMR1 - Lane 3 purified protein, C-MR2 - Lane 1 purified protein, CD209 - Lane 3 purified protein, C-lectoxin - Lane 2 purified protein, and LPB - Lane 1 purified protein and CD63 - Lane 2 purified protein. M - Protein marker with KDa.


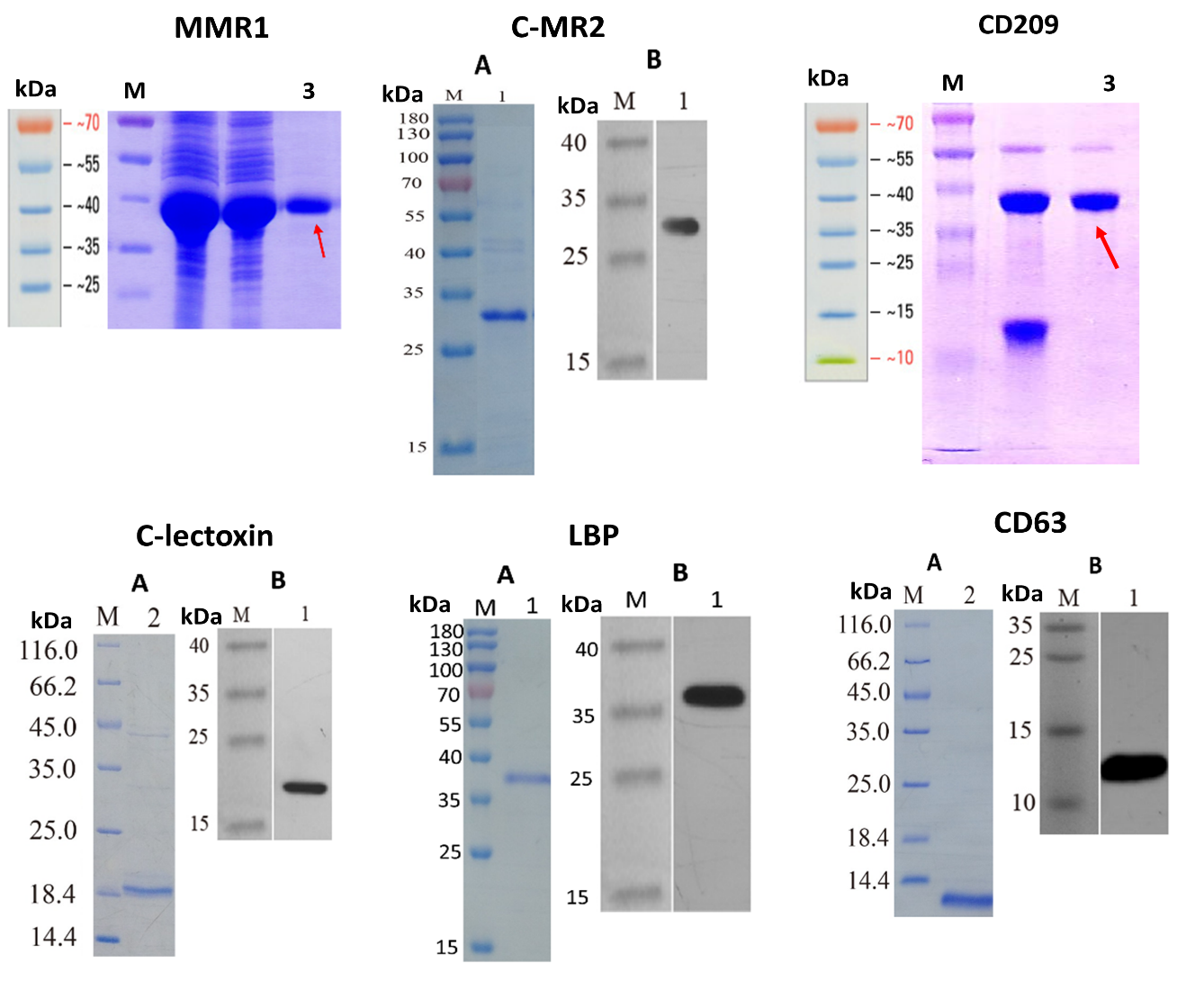


**Figure S8**. SPR sensograms showing association and dissociation curves generated during multiple analyte concentration injections.


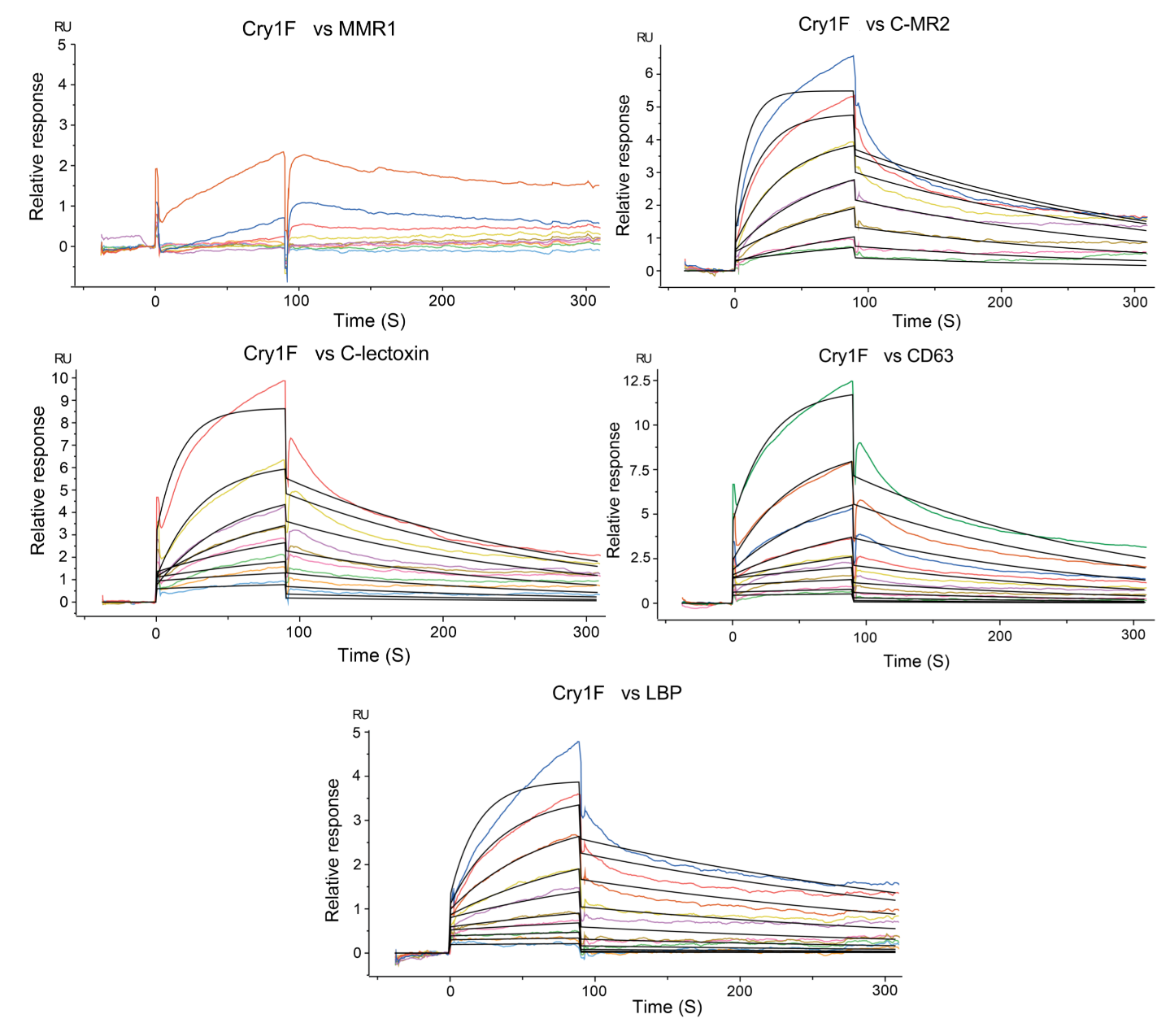


**Figure S9.** Lack of binding between CD209 and Cry1F as evidence by SPR sensogram (A) and pull-down assays (B). Each panel in (B) presents SDS-PAGE gels initially stained with FastBlue Protein stain (left) and further stained using ProteoSilver silver staining kit (right). Lanes M - TrueColor protein marker, Lanes 1 - Bait flowthrough, Lanes 2 - Prey flow through, Lanes 3 - Final elution, Lanes 4 - Purified bait protein, Lanes 5 - Prey protein (activated Cry1F) and Lanes 6 - Non-treated control. Lanes 3 – Eluted protein sample


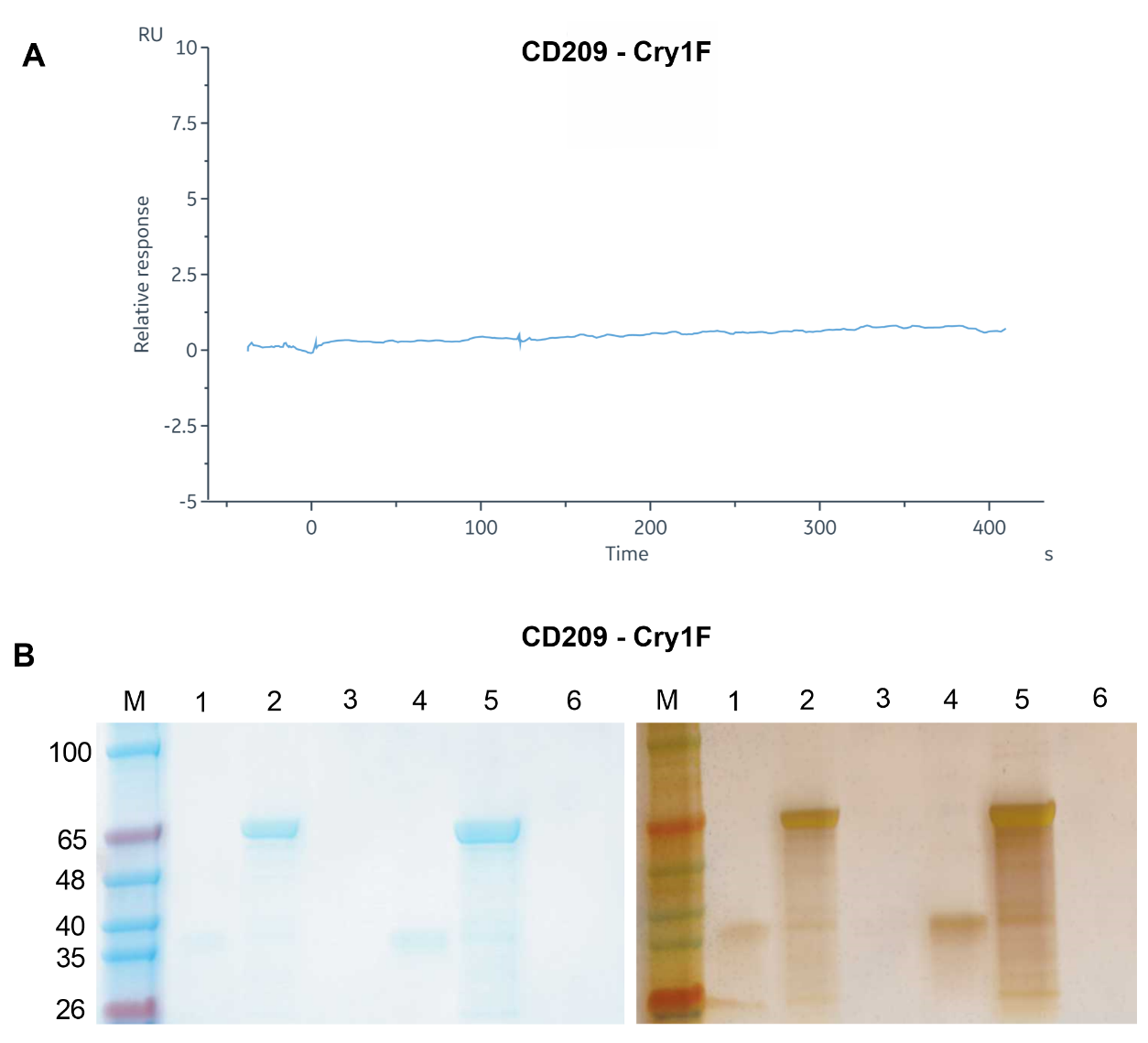


**Table S2.** Primers used to amplify immune genes from midgut tissue.

| **S. no** | **Gene names** | **Primer sequences (5’ – 3’)** | **NCBI Acc. No.** |
| --- | --- | --- | --- |
| 1 | MMR1 | FP - ATGTTTCGACAAACGTTGTTTATTTCTC  RP - TTACTCATCATCTCCAAATTTAAAATCC | XM_028314985 |
| 2 | C-MR2 | FP - ATGAAAGTCTCTATTATTCTAGTTTTGG  RP - CTAAACTAGATCAGCTTTCTTTTCGC | XM_028308123 |
| 3 | LBP | FP - ATGGAGCGATTCGCTGTTTTCATAC  RP - TCACTCGTTTCTCTCTTTCTCGCAT | XM_028302111 |
| 4 | CD63 | FP - ATGCTATCGAAAATATTTACAACAGTG  RP - TCAAGGATATGCTCCATGCTCCTC | XM_028322859 |


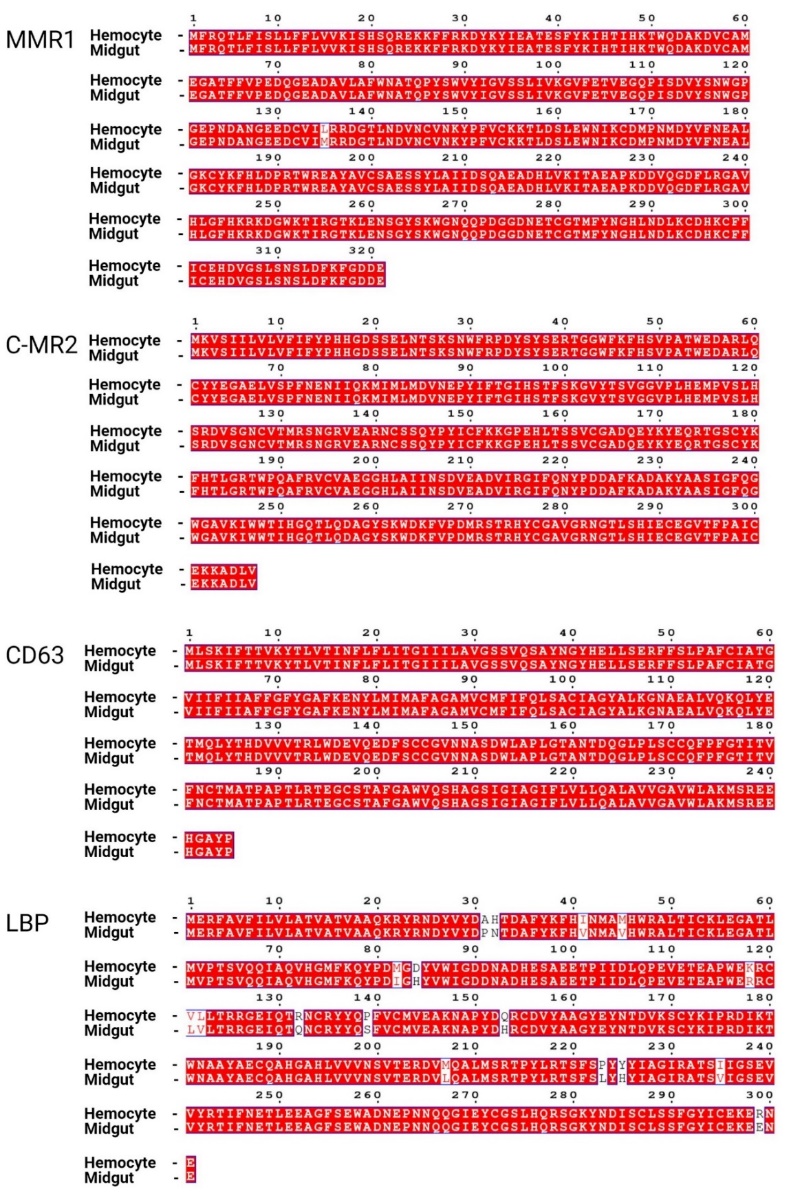
**Figure S10.** Protein sequence alignment (ESPript v 3.0) of immune-related genes MMR1, C-MR2, CD63 and LBP obtained from hemocyte and midgut tissue.
